# Electrochemical Amino Acid Sensing: A Review on Challenges and Achievements

**DOI:** 10.3390/bios11120502

**Published:** 2021-12-07

**Authors:** Kaveh Moulaee, Giovanni Neri

**Affiliations:** 1 Department of Engineering, University of Messina, C.Da Di Dio, I-98166 Messina, Italy; kaveh.moulaee@unime.it; 2 Center of Excellence in Electrochemistry, School of Chemistry, College of Science, University of Tehran, Tehran 16846-13114, Iran

**Keywords:** amino acids, electrochemical sensors, cysteine, methionine, tryptophan, tyrosine, histidine

## Abstract

The rapid growth of research in electrochemistry in the last decade has resulted in a significant advancement in exploiting electrochemical strategies for assessing biological substances. Among these, amino acids are of utmost interest due to their key role in human health. Indeed, an unbalanced amino acid level is the origin of several metabolic and genetic diseases, which has led to a great need for effective and reliable evaluation methods. This review is an effort to summarize and present both challenges and achievements in electrochemical amino acid sensing from the last decade (from 2010 onwards) to show where limitations and advantages stem from. In this review, we place special emphasis on five well-known electroactive amino acids, namely cysteine, tyrosine, tryptophan, methionine and histidine. The recent research and achievements in this area and significant performance metrics of the proposed electrochemical sensors, including the limit of detection, sensitivity, stability, linear dynamic range(s) and applicability in real sample analysis, are summarized and presented in separate sections. More than 400 recent scientific studies were included in this review to portray a rich set of ideas and exemplify the capabilities of the electrochemical strategies to detect these essential biomolecules at trace and even ultra-trace levels. Finally, we discuss, in the last section, the remaining issues and the opportunities to push the boundaries of our knowledge in amino acid electrochemistry even further.

## 1. Introduction

**Figure 1:**
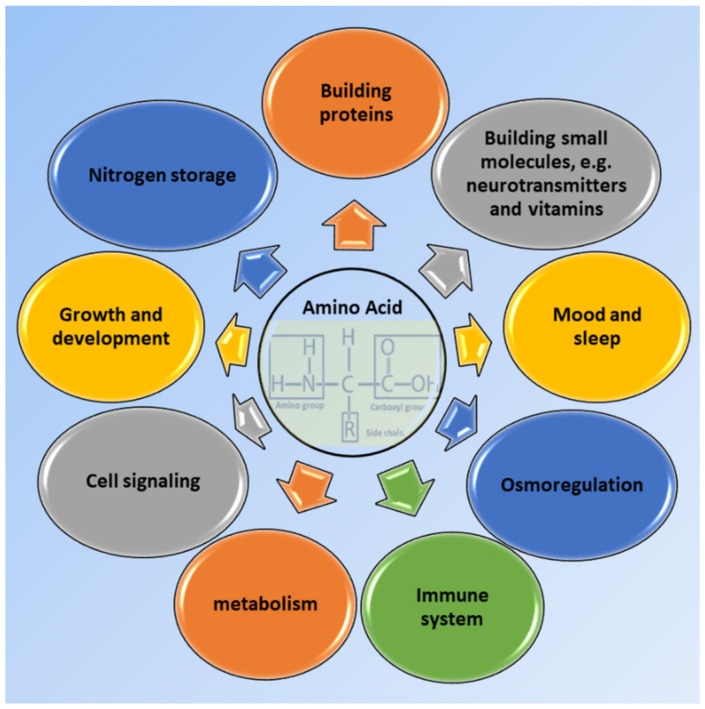
Some crucial roles of amino acids affecting our body functions.

Small molecules and macromolecules are commonly regarded as building blocks of known life. Carbohydrates, proteins, nucleic acids and lipids are four main subgroups of macromolecules, whereas amino acids, hormones, vitamins, neurotransmitters and metabolites, as well as numerous drugs, are common examples of small molecules. In our body, there are thousands of proteins made up of 20 different amino acids which contain an amino group and a carboxyl group at each terminal [1]. Nine amino acids, i.e., methionine, tryptophan, histidine, phenylalanine, valine, threonine, lysine, leucine and isoleucine are called essential amino acids, as they cannot be synthesized endogenously in the human body. On the other hand, our body can synthesis the rest, known as non-essential amino acids, either from glucose, e.g., glycine, glutamate, glutamine, alanine, aspartate, arginine, asparagine, proline and serine, or from the metabolism of other amino acids, e.g., tyrosine from phenylalanine and cysteine from methionine [2]. These amino acids are building blocks and essential elements for synthesizing a large number of low-molecular-weight substances such as glutathione, thyroid hormones, creatine, melatonin, serotonin, melanin and heme, whose importance for body function is well-established [3]. The typical level of amino acids in plasma is in the micromolar range. For instance, the concentration of aspartic acid in plasma can be as low as 2–11 µM, while glutamine can be found in plasma at levels as high as 352–689 µM [4]. Some inherited metabolic disorders like phenylketonuria, tyrosinaemia and hyperglycinaemia can alter the level of amino acids in the body. Elevated levels of total plasma amino acid can be assigned to fructose intolerance, kidney failure and ketoacidosis. On the contrary, lowered level of total amino acids plasma can be a sign of nephrotic syndrome, Huntington’s disease, rheumatoid arthritis and fever. Figure 1 presents a general schematic view of some of the most vital functions of amino acids in our body.

Nowadays, the evaluation of the amino acids level in biological media, e.g., in blood, sweat, urine and saliva, is inspiring new approaches in the prevention and treatment of metabolic disorders such as diabetes, obesity and cardiovascular disorders, as well as infectious disease (including viral diseases), neurological dysfunction and infertility [4,5,6]. Table 1 summarizes some of the many crucial functions of five electroactive amino acids in both living organisms and industrial world.

Due to the great nutritional, biotechnological and clinical significance of amino acids, a substantial part of research is being directed to develop effective and reliable analytical protocols for evaluating amino acids. Therefore, a wide variety of analytical strategies such as near infrared [23] and Raman spectroscopy [24], UV-Vis spectroscopy [25], surface-enhanced Raman spectroscopy [26], electrochemiluminescence [27], tandem mass spectroscopy [28] and electroanalytical methods [29] have been developed for the detection of amino acids. However, the lack of a strong chromophore hampers the direct detection of amino acids by using UV-Vis or fluorescence spectroscopy, and, hence, target amino acids should be converted into the chromophore-containing derivatives before using these methods [30]. Clearly, detection strategies that do not require derivatisation step(s) are strongly preferred, in terms of both cost and simplicity, over time-consuming, complicated and costly methods involving derivatisation protocols. Regarding this, electrochemical methods can offer a great advantage over other analytical protocols for obviating the necessity of derivatisation steps. However, the electrochemical analysis of amino acids, like all other analytical methods, has its limitations and drawbacks, which will be also addressed in this review.

Nowadays, clinical diagnoses are no longer carried out solely in clinical laboratories. Instead, they are routinely performed in several settings such as hospital point-of-care settings or by caregivers out of hospital and even by patients themselves at home [31,32]. Electrochemical methods are ideally well-adjusted for these emerging nonhospital analyses. Electrochemical sensing strategies potentially offer the simple, fast, cost-effective, sensitive and, to some extent, selective detection of bioanalytes relevant to clinical diagnostic tests and represent promising alternatives for common clinical methodologies [33]. To date, a wide spectrum of methods has been developed for signal transduction and target detection, which allows electrochemical techniques to be used as effective sensing protocols. Therefore, electrochemical sensing platforms are leaving the field of laboratory research and successfully stepping forward to the point-of-care detection of biomolecules such as lactate and glucose that mostly rely on bioaffinity recognition and electrochemical transduction approaches. 

**Figure 2:**
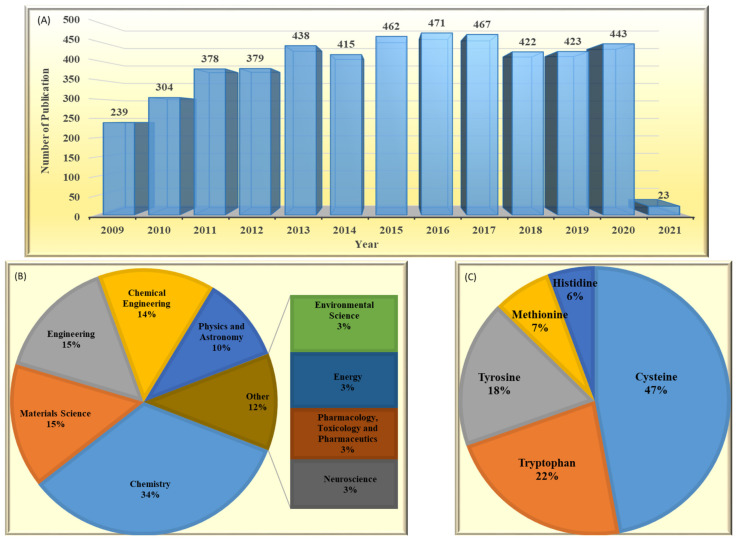
A comparative demonstration of (**A**) published papers per year searching ‘amino acid’ and ‘electrode’. (**B**) Categorized published papers in the first 10 scientific areas according to number of papers. (**C**) Published papers for 5 well-known electroactive amino acids searching the ‘name of the amino acid’ and ‘electrode’ and ‘detection’. All results refer to a search in the Scopus database (9 January 2021) by limiting the search to the title, abstract and keywords (Title, Abs and Key).

Even glimpsing at the well-known literature databases will reveal that the electrochemistry of amino acids is a dynamic ongoing subject that encompasses many scientific fields from biochemistry and genetics to chemistry and even engineering areas. Figure 2 presents the results of a literature survey using the Scopus database. It is seen from the number of published papers (since 2010) concerning the electrochemistry of amino acids that research in this field is still appealing and being actively perused. Scientists in different fields such as chemistry, material science and engineering are actively investigating the electrochemistry of amino acids to fulfill their desired aims. It is frequently highlighted that there are five well-known electroactive amino acids [34,35,36,37], including cysteine, tryptophan, tyrosine, methionine and histidine, that have been the core of many research activities in the last decade. Of these, as can be seen in Figure 2C, cysteine has been the subject of about half of all published papers. In the following sections, we will discuss the importance of this amino acid as a benchmark in amino acid electrochemistry.

This review aims at acquainting the readers with different aspects of amino acid sensors based on electrochemical strategies. For this, we will start with a discussion on amino acid electroactivity to provide a general perspective for readers on the electrochemistry of amino acids. After that, in the following sections, we will go further into details and evaluate the proposed electroanalytical strategies, to date, for five well-known electroactive amino acids, namely cysteine, tyrosine, tryptophan, methionine and histidine. Throughout these sections, significant performance metrics including the limit of detection, linear dynamic range(s) and, sensitivity and durability of the proposed electrochemical sensors will be presented. Finally, in the last section, conclusions and outlooks, the main ongoing challenges and difficulties in the electrochemistry of amino acids that should be addressed to pave the way for future achievements are discussed, and possible guidelines are presented.

## 2. Amino Acid Electroactivity

The attractiveness of electroanalytical techniques lies greatly in their directness. In other words, despite most of the spectroscopic methods for the evaluation of amino acids that require derivatisation, the proposed electroanalytical methods are mostly label-free. Moreover, it is worthwhile to mention that, since the electrochemical oxidation of both free and bounded amino acids, e.g., in enzymes and proteins, exhibit similar voltammetric profiles [37], electrochemical methods can be used in proteomics for proteins that contain at least one electroactive amino acid or another electroactive centre. As an example, Oliveira-Brett’s group [38] reported that different amyloid beta peptides related to Alzheimer’s disease were successfully evaluated thanks to the presence of five electroactive amino acids, in the structure of these peptides, i.e., one tyrosine, three histidines and one methionine. They reported that, depending on the length and content of these amyloid beta peptides, one or two oxidation peaks are observed. They ascribed the first peak to the electrooxidation of tyrosine residue and the second peak to the presence of both methionine and histidines residues. However, there are some drawbacks, like all other analytical methods, to be addressed for the electroanalysis of amino acids.

Despite the prominent merits of electrochemical methods, the electrochemical signal of d-amino acids and their l-isomer are both often indistinguishable, and discriminating between them is hard to achieve with bare electrodes. One serious concern is that, for electroactive amino acids, signal overlapping and/or large overpotential are still challenging. For example, the oxidation peaks of tyrosine and tryptophan overlap at the most common bare electrodes, or the oxidation of histidine and methionine in aqueous solution occurs at relatively high potentials, usually >1 V vs. Ag/AgCl reference electrode. In fact, even now, there are some major challenges that need to be thought about before using electrochemical sensors, beyond their current use, in practical application in point-of-care diagnostics. Nonetheless, the unprecedented efforts are being devoted in this field, especially to develop new sensing materials, hold great promise that this aim will be fulfilled in the upcoming years.

**Figure 3:**
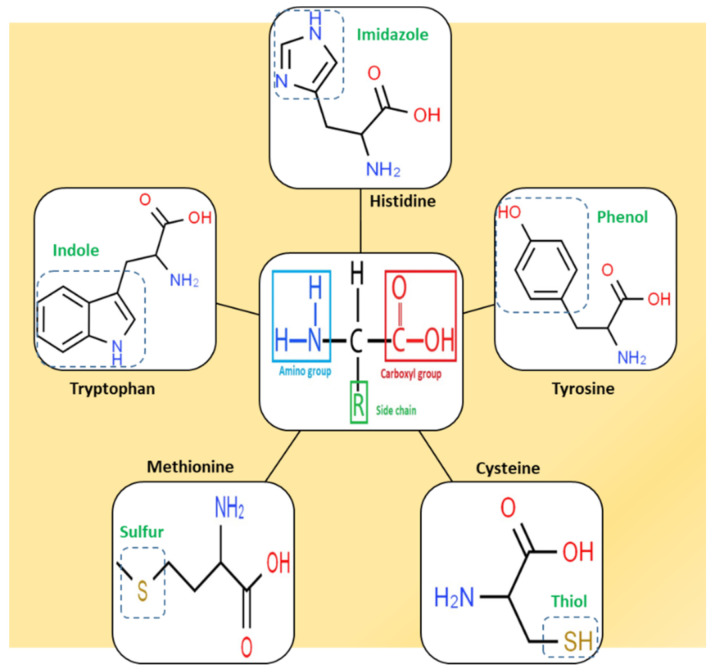
Chemical structure of the five well-known electroactive amino acids highlighting their electroactive side chain.

Another interesting subject in electrochemistry of amino acids is the evaluation of the possible oxidation pathway(s) by which amino acids participate in electrochemical reactions at the electrode surface. It is frequently reported in the literature that the electrooxidation of amino acids is often an irreversible, complicated and multistep phenomenon. Even though the reaction mechanism of amino acids at the electrode surface is still a controversial issue, it is usually assumed that, at first, amino acids are adsorbed onto the electrode via their carboxyl group facilitating electron transfer between the electrode and electroactive part of amino acids. These electroactive parts of amino acids are located in the side chain of amino acids [38]. For instance, the electroactivity of cysteine, tyrosine and tryptophan is assigned to a thiol, phenol and indole function, respectively, which exist in the side chain of these amino acids (Figure 3). Methionine and histidine are other electroactive amino acids whose electroactivity is ascribed to the sulphur- and imidazole-containing side chain, respectively.

**Figure 4:**
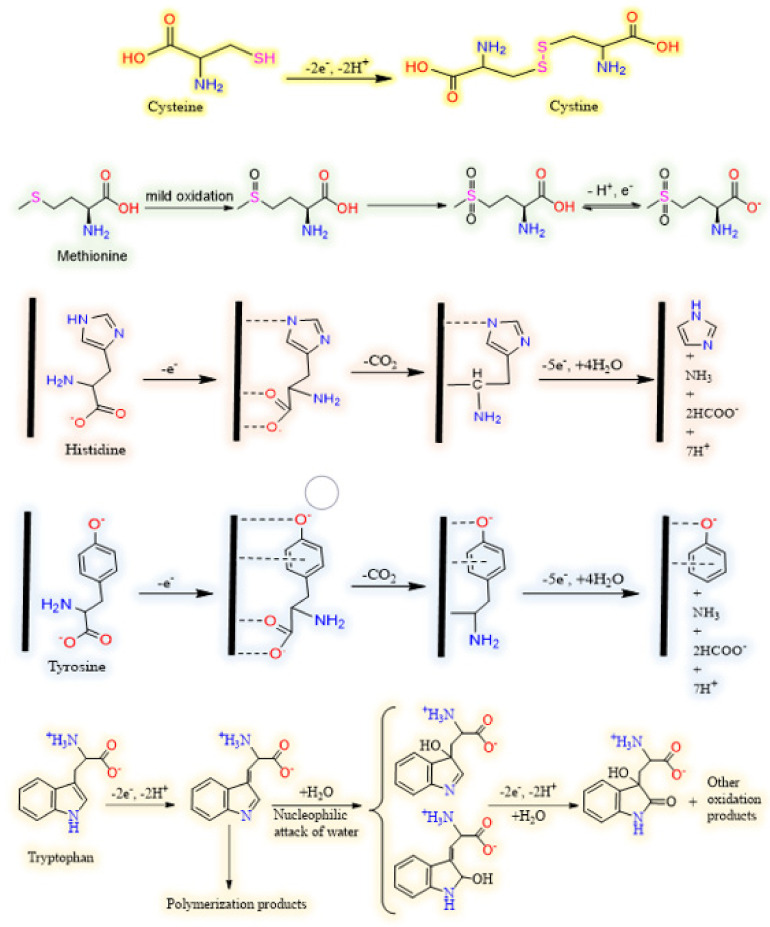
Schematic representations of typical electrooxidation pathway for cysteine, methionine, histidine, tyrosine and tryptophan (from up to down, respectively).

There is a big controversy among researchers over the electrooxidation mechanism of amino acids. This, in the authors’ opinion, stems from the high susceptibility of electrode reaction pathways to the reaction conditions, e.g., type and concentration of supporting electrolyte, pH, type of electrode and even amino acid concentration itself. This high dependency of amino acid electrochemistry on experimental conditions is a consequence of their structural features, since amino group, carboxyl group and mostly even the functional groups presented in the side chain are very liable to variation in measurement conditions. For example, variations in pH and electrolyte might affect the reaction pathway through changes in the type/density of the surface charge, the degree of solvation and the adsorption/desorption properties of amino acids. Figure 4 is drawn based on the general electrooxidation pathways reported in the literature for cysteine [39,40], methionine [41], tryptophan [42,43], tyrosine and histidine [37,44]. Since the main goal of this review is to collect and present recent findings concerning quantitative electrochemistry of amino acids, we inevitably have to skip going further into details. However, we strongly encourage the interested readers to see these qualitative studies as the basis for better understanding the current issues and to innovate new approaches to address them. 

## 3. Electrochemical Analysis of Amino Acids

### 3.1. Sulphur Containing Amino Acids

#### 3.1.1. Cysteine

**Figure 5:**
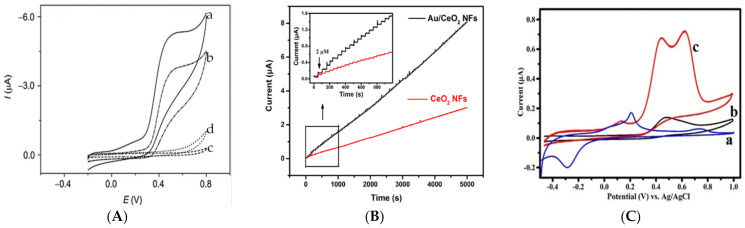
(**A**) Cyclic voltammograms of 0.5 mmol/L (a, c and d) and 0.2 mmol/L (b) cysteine at an Au/Nafion/GCE (a, b), Nafion/GCE (c) and bare GCE (d), Scan rate: 20 mV/s in PBS (pH 2.0) [65]. (**B**) Amperometric response curves of the CeO_2_ NFs and Au/CeO_2_ NFs modified SPCEs in 0.01 M PBS (pH 7.4) in the concentration range of 2.0–200 μM (applied potential 0.7 V) [102]. (**C**) Cyclic voltammograms of (a) Ag/ITO, (b) Poly dopamine /ITO and (c) Ag-Poly dopamine/ITO in 0.1 M PBS (pH = 5.0) solution containing 25 μM cysteine at scan rate of 50 mV/s [68]. Copyright 2021 Royal Society of Chemistry.

Protein electrochemistry was born in the middle of 20th century, somehow, thanks to cysteine electroactivity when Heyrovsky invented polarography and observed a so-called ‘pre-sodium wave’ [45]. Later, it was concluded that the pre-sodium wave is caused by the cysteine residue of under-study proteins. Since then, cysteine, among five electroactive amino acids, has been a continuously appealing subject for researchers, such that ca. 50% of all developed electrochemical amino acid sensors has been reported for cysteine; see Figure 5C. It has been pointed out that human blood plasma contains three forms of cysteine, including free cysteine, cystine and protein-bound cystine. Cysteine is a thiol, while the latter two are in disulphide form [46]. The normal level of free cysteine in a healthy individual is reported to be in the range of 5–30 μM for blood plasma [47], while the urinary excretion of cysteine is in the range of 3–33 mg/24 h [48]. Therefore, to achieve the desired sensitivity to detect cysteine in these low concentration ranges, diverse modifiers have been proposed to be exploited in electrochemical cysteine sensors. Noble metals, e.g., gold [49,50,51,52,53,54,55,56,57,58,59,60,61,62,63,64,65,66], palladium [67], platinum [67], silver [68,69,70], noble metal composites, e.g., gold/copper [71], gold/silver [72], gold/nickel [73], gold/platinum [74] and silver/palladium [75], have been extensively used. Additionally, metal oxides, e.g., CeO_2_ [76], Cu_(X)_O [77], Fe_2_O_3_ [78], MgO [79], MnO_2_ [80], NiO [81], SnO_2_ [82], TiO_2_ [83], WO_3_ [84], Y_2_O_3_ [85] and ZnO [86], as well as organic modifiers [87,88,89,90,91,92,93,94,95,96,97,98,99,100], have also been explored as potential alternatives for noble metals. 

It is well-known that the side chain sulphur of cysteine has remarkable affinity for gold, silver and copper in order of Au > Cu ˃ Ag [101]. This has encouraged many researchers to explore the capability of these metals to design more effective cysteine sensors. Figure 5 illustrates the intense effect of these metals on enhancing the peak currents and, consequently, the sensitivity of the proposed modified electrodes compared to the bare ones.

Wang et al. [65] investigated the effect of Au NPs on cysteine electroanalysis, Figure 5A, and no detectable peak was observed for cysteine at bare GCE and Nafion/GCE (Figure 5A, d and c). However, after adding gold NPs to the electrode, an oxidation peak emerged whose peak current increased upon increasing cysteine concentration (Figure 5A, b and a). Interestingly, sensor performance was explored in acidic (pH = 2) and neutral (pH = 7) solutions, and they reported that, in acidic medium, narrower linear range (3.0–50 µM) and higher sensitivity (22.7 µA/mM L^−1^) are obtained compared with neutral pH (2.0–80 µM and 4.08 µA/mM L^−1^). Additionally, the interference effect of ascorbic acid and uric acid were found to be more effectively suppressed in neutral pH. Figure 5B clearly shows that the addition of Au nanoparticles to the as-prepared cerium oxide nanofibers (CeO_2_ NFs) results in a notable promotion in the sensitivity (2.7 times) of the proposed amperometric cysteine sensor. Using this composite, Au/CeO_2_ NFs, Cao et al. [102] developed an ultra-sensitive cysteine whose LOD was as low as 10 nM. Despite the substantial effect of gold, finding a less-expensive material, e.g., silver, to undertake the role of gold is an active area for researchers due to the prohibitive cost of gold. Since cysteine showed no electroactivity at an indium tin oxide (ITO) electrode, Thota et al. [68] modified the bare ITO with poly dopamine and silver nanoparticles. They found a remarkable promotion in peak current for this modified electrode (Ag-polydopamine/ITO), wherein both Ag/ITO and polydopamine/ITO showed significantly lower peak currents (Figure 5C). This is a valuable example of the synergistic effect of polydopamine and silver nanoparticles on the cysteine electrochemical signal. Moreover, two oxidation peaks were observed at Ag-polydopamine/ITO during the forward potential sweep (Figure 5C) that are likely due to the presence of two different catalytic sites on the electrode modified for cysteine oxidation. This sensor exhibited a linear range of 0.05–300 µM, and the LOD was calculated to be 0.02 µM. In spite of being both noble and precious metals, just a few reports have found that used palladium [67] or platinum [103,104] nanoparticles for electrochemical cysteine determination.

**Figure 6:**
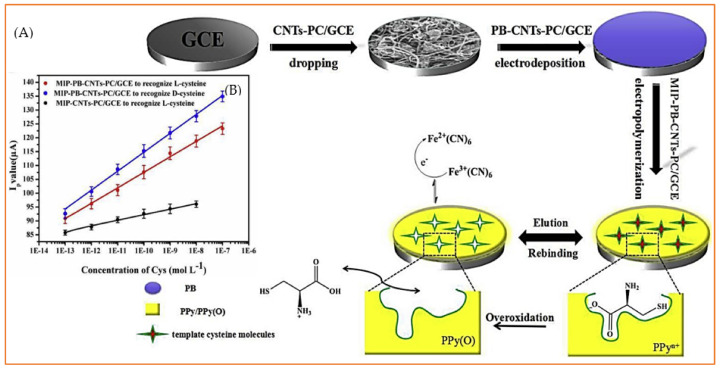
(**A**) The preparation process for a MIP-PB-PC-CNTs/GCE sensor. (**B**) The relationship of the peak values of MIP-PB-CNTs-PC/GCE and MIP-CNTs-PC/GCE with the concentration of l-cysteine and d-cysteine. Reprinted with permission from ref. [112]. Copyright 2019 Elsevier.

In addition to noble metals, copper compounds, in either oxide [77,105], sulphide [106] or other complex forms [107,108,109,110,111] have also been interesting options for researchers in this field owing to the high affinity of copper for the sulphur moiety of cysteine. In an interesting study, Li et al. [105] synthesized an octahedral Cu_2_O/polystyrene composite and, consequently, removed the styrene from the Cu_2_O lattice easily by treating the Cu_2_O/polystyrene composite with tetrahydrofuran to obtain hollow cubic Cu_2_O particles. They showed that, generally, the presence of Cu_2_O particles promotes the sensitivity of the sensor, compared to bare GCE, and more importantly, the electrochemical response of cysteine significantly improves (about 2.8 times) for hollow cubic Cu_2_O particles with respect to the Cu_2_O/polystyrene composite (Figure 6A). Linear measurement ranges were found to be 6.0–100 µM and 0.5–200 µM for Cu_2_O/polystyrene and hollow cubic Cu_2_O particles, respectively, whereas the LOD for the latter (0.14 µM) was about 16 times lower than the first one (2.3 µM). This revealed that both the nature and structure of modifier should be wisely tuned to reach the optimum performance for this sensor. Iron compounds, after copper ones, are by far the most studied non-noble-metal-based modifiers in cysteine electroanalysis [56,78,112,113,114,115,116,117,118,119,120,121,122]. Duan et al. [112] developed an outstanding sensor taking advantage of extreme stability, excellent electrocatalytic activity and the redox properties of Prussian blue (PB, Fe_4_[Fe(CN)_6_]_3_). To build this sensor (Figure 6A), metal organic-framework-derived porous carbon (PC) was first synthesized, mixed ultrasonically with carbon nanotubes (CNT) and drop-cast on the GCE surface. PB was electrodeposited on CNTs-PC/GCE, and then, this electrode, PB-CNTs-PC/GCE, was transferred to a solution containing both pyrrole and cysteine to electropolymerize pyrrole in the presence of cysteine template. Here polypyrrole works as a molecularly imprinted polymer (MIP). In the last step, the MIP-PB-CNTs-PC/GCE electrode was immersed in PBS, and cysteine templates were removed from the polypyrrole structure through an overoxidation process to leave imprinted cavities behind. The extraordinary selectivity and sensitivity of this sensor are closely related to the presence of imprinted cavities and Prussian blue, respectively. This sensor was very selective so that it could differentiate even d-cysteine from l-cysteine and detect the l-isomer in a linear range of 10^−13^–10^−7^ mol L^−1^ with LOD as low as 6.0 × 10^−15^ mol L^−1^. Figure 6B clearly exhibits the remarkable effect of PB on the sensitivity of the sensor, wherein slope of calibration curve is much lower in the absence of PB. Though the preparation process of this sensor was rather complex (Figure 6A), the resultant sensor was extremely sensitive and selective. In another approach, Zhou et al. [113] proposed a magnetic GCE modified with a Fe_2_O_3_/polydopamine/Cu_2_O composite to detect cysteine and reported an ultra-sensitive sensor with an outstanding LOD of 83.0 pM (83.0 × 10^−15^ mol L^−1^). In contrast to the work of Duan et al. [112], in this sensor, the iron oxide moiety is used, mainly because of its magnetic properties, and iron oxide is not directly involved in electrochemical signal production because Cu^2+^ ions undertake this task.

**Figure 7:**
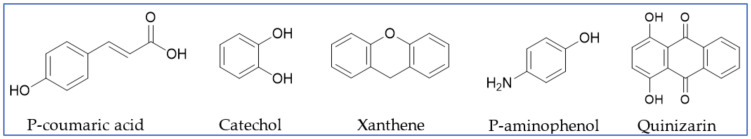
Benzene-derivative organic modifiers used in electrochemical cysteine sensors.

Although a big share of developed cysteine sensors exploit metal-based modifiers, some valuable reports are found in which merely organic materials serve as modifiers. These organic modifiers for electrochemical cysteine detection are mainly either simple carbon structures, e.g., carbon nanofiber [87,99], carbon black [90], nanocarbon [94] and ordered mesoporous carbon [95], or benzene-derivative compounds, e.g., *p*-coumaric acid [88], catechol [89,92,93,96], xanthene [123], *p*-aminophenol [98] and quinizarin [100]; see Figure 7. 

It has widely been accepted that the first category members, carbonaceous materials, enhance the performance of sensors mainly through improving the electrode surface features such as specific surface area and conductivity, while the latter ones, benzene-derivative compounds, promote the function of sensors through mediating the electron transfer between cysteine and electrode as electrocatalyst. It is noteworthy to mention that these mediators not only bring about a great enhancement in sensitivity by electron shuttling between cysteine and electrode but also may effectively improve the selectivity of the sensor. Interesting works have been reported by Compton’s group wherein they selectively measured cysteine and homocysteine in the presence of ascorbic acid and glutathione [94] or homocysteine in a medium containing cysteine ascorbic acid and glutathione [92]. Table 2 summarizes the reported works, from 2010 to now, in the field of cysteine electrochemical detection, and the most important related figures of merit to give readers the recent orientations and achievements in this field.

#### 3.1.2. Methionine

**Figure 8:**
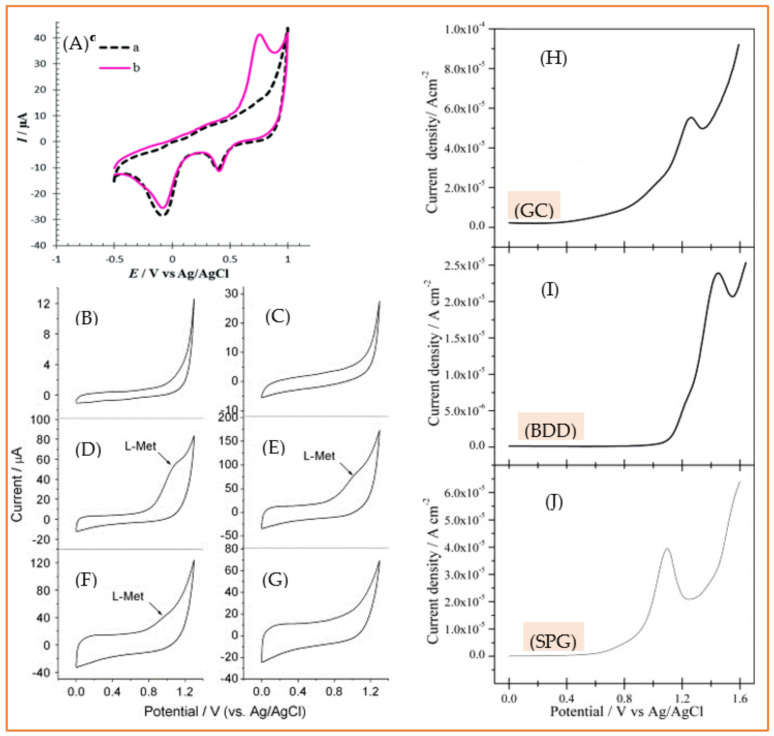
(**A**) Cyclic voltammograms of Ru/Pt-modified electrode (**A**) without and (**B**) with 100 μM methionine in 0.1 M phosphate buffer solution (pH = 7), with a scan rate 10 mV s^−1^. Reprinted with permission from ref. [184]. Copyright 2017 Royal Society of Chemistry. CVs of 5 mM methionine in pH 5.5 PBS at bare GC (**B**) and GO/GC electrodes pretreated at different potentials. (**C**): −0.65 V; (**D**): −0.75 V; (**E**): −0.8 V; (**F**): −0.9 V; (**G**): −1.2 V, Reprinted with permission from ref. [195]. Copyright 2011 Elsevier. Comparison of the DPV of different carbon-based electrodes: GC (**H**), BDD (**I**) and SPG (**J**) electrodes for the direct oxidation of 1 mM methionine in 0.1 M phosphate buffer solution (pH 7). Reprinted with permission from ref. [196]. Copyright 2014 Elsevier.

Methionine, unlike cysteine, is an essential amino acid in human and other animals as it is not synthesized in the body, and the needed methionine should enter the body through methionine-containing foods or supplements. The literature survey showed that, in spite of the importance of methionine in body function, far fewer reports are found on the electrochemical detection of methionine in comparison to cysteine. This may originate from the lower electroactivity of the sulphur group of methionine that usually results in, if any, an ill-defined oxidation peak with high over-potential in aqueous solution (see Figure 8). Methionine occurs in our biological fluids at almost the same levels as cysteine. For example, for a healthy individual, the concentration of methionine in the blood serum is, on average, 25.5 µM (the range can be 13.7–43.5 µM) and in the urine sample is, on average, 5.9 µM (the range can be 0.4–35.1 µM) [180]. Therefore, to be applicable in biological-sample analysis, methionine sensors should practically have almost the same sensitivity as cysteine sensors, though the sulphur moiety of methionine is not as electroactive as the cysteine one. Nevertheless, some valuable studies have been carried out to face this challenge.

Noble metals and their alloys, as expected, are the main modifiers used in electrochemical methionine sensors [181,182,183,184]. These noble metals are exploited either in monometallic form [181,184] or as a bimetallic compound, e.g., Ru/Pt [182] and Ag/Au [183]. Bimetallic modifiers are expected to surpass their monometallic counterparts through improving the effective surface area, electron transfer rate, biocompatibility, electrocatalytic activity and invulnerability against interfering species and/or intermediate by-products of electrochemical reactions. In a study, Tavakkoli et al. [182] deposited a bimetallic monolayer of ruthenium/platinum (Ru/Pt) on gold electrodes. For this, first, the underpotential deposition of copper on gold electrode was carried out and followed by the replacement of this copper layer with Ru and Pt at open-circuit potential. This method results in an ultra-thin Ru/Pt coating on the gold electrode that effectively facilitates the electrooxidation of methionine, as is seen in Figure 8A. A wide linear measurement range, 0.006–102 µM and a low LOD of 2.0 nM, were reported for this electrochemical sensor. Apart from precious noble metals, other more earth-abundant and cost-effective metal compounds such as MoS_2_ [185], ZnO [186], MnO_3_ [187], NiO [188], TiO_2_ [189], Cu(II) phthalocyanine [190] and cobalt hydroxide nanoparticles [191] have shown acceptable performance as well.

Turning our attention to organic modifiers, some interesting reports are found in which a member of the carbon allotrope family, e.g., graphene derivatives, carbon nanotubes or diamond, is the key player [192,193,194,195]. Regardless of being eco-friendly, cost-effective and biocompatible, carbon-based modifiers owe a big part of their importance to the facility of surface and bulk modification in these materials. For example, the nature and abundance of oxygen functional groups on graphene oxide can be easily altered by applying different reduction potential, as Zhang et al. reported [193].

As shown in Figure 8B, no oxidation peak for methionine is observed in the cyclic voltammograms of bare glassy carbon electrodes (GCE). After adding graphene oxide (GO) to the GCE, different pretreatment potentials were applied to convert GO to reduced graphene oxide (rGO). The reduction of GO to rGO results in an increase in conductivity by removing the oxygen factional groups from GO. This has two contradictory effects on the electrochemical performance of the sensor. On one hand, converting GO to rGO is beneficial since rGO is much more conductive compared to GO. On the other hand, the presence of these functional groups is critical, as they play the key role in the electrooxidation of methionine. Considering this, the best performance for this sensor is observed when an intermediate reduction potential is applied in the conversion of GO to rGO (Figure 8C). This example clearly shows that how much an electrochemical sensor can be susceptible to the surface features insofar as even 0.1 V variation in applied potential can result in a completely different response.

Regardless of all modification strategies, Gómez-Mingo et al. [194] compared the performance of three well-known bare electrodes, namely glassy carbon (GC), boron doped diamond (BDD) and screen-printed graphite (SPG) electrodes, as electrochemical sensors using differential pulse voltammetry (Figure 8H–J). No chemical or electrochemical treatment was carried out to activate the bare electrodes where the SPG electrode showed the best performance, taking into account both lower oxidation peak potential and higher peak current. Although the linear dynamic range (50–5000 µM) and LOD (95 µM) were not appropriate enough to be used for measuring methionine in biological fluids, this simple electrochemical sensor can be successfully used to evaluate methionine in pharmaceutical samples. Table 3 presents various developed strategies for electrochemical methionine detection over the last decade.

### 3.2. Aromatic Amino Acids

#### 3.2.1. Tryptophan

The recommended daily dose of tryptophan (Trp) for a healthy adult is estimated to be in the range of 250–425 mg. This means that, since Trp is an essential dietary requirement for human and cannot be synthesized in the body, a dietary intake of approximately 3.5–6.0 mg/kg of body weight should be daily provided through foods, supplements or medicines [14]. Of this dietary Trp, less the 1% enters through the protein synthesis pathway, while the rest is degraded and used to produce some physiologically significant substances such as melatonin, serotonin, tryptamine, niacin and kynurenine. Clinical relevant urinary Trp in a healthy adult is in the range of 20–70 μM. It is worth mentioning that, due to the fluctuation in substance concentration in urine samples with time of day and intake of water, it is more acceptable to report total 24-h amount rather than the measured concentrations at any time. However, a 24-h urine collection is not convenient. This normal range of urinary Trp can even increase to 0.1–10 mM in people suffering from some inborn metabolic diseases [201]. Likewise, abnormalities in the blood level of Trp, which is in the range of 45.5–83.1 μM for a healthy individual, can also be a sign of the body malfunctioning [202]. Considering the biologically significance and widespread usage in food and feed industries, and of course its relatively high electroactivity, tryptophan has acquired the second place, after cysteine, with respect to the number of published paper in this field; see Figure 2C.

**Figure 9:**
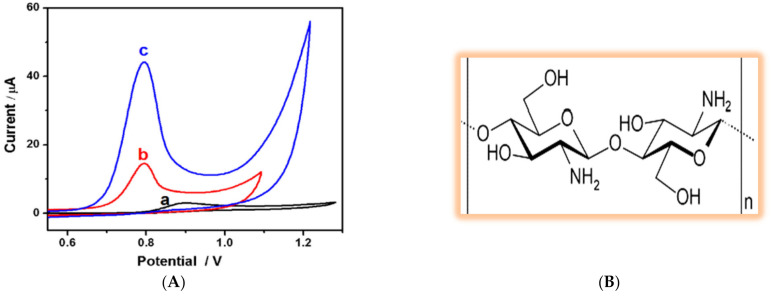
(**A**) Cyclic voltammograms of 100 µM tryptophan (in 0.1 M PBS pH 4.0) at (a) bare GCE, (b) electrochemically activated bare GCE and (c) chitosan-modified GCE. Reprinted with permission from ref. [42]. Copyright 2015 Elsevier. (**B**) The chemical structure of chitosan.

In contrast to cysteine and methionine, noble metals are not at top of the list of the most-used modifiers for electrochemical Trp sensors; this place is taken by organic modifiers. Designing these organic-based electrochemical sensors can be as easy as adding MWCNTs to a carbon paste electrode [203] or blending carbon dots with chitosan [204], or it might require a more sophisticated process, e.g., the functionalization of graphene with poly(sodium 4-styrenesulphonate) [205], carbon black with oxygenated groups [206] or graphene quantum dots with amino groups [207]. In a very simple approach, Zanini et al. [42] modified an electrochemically activated GCE by dipping it in a solution containing 0.5 wt% chitosan for 20 min. Though the preparation process was quite easy, this electrochemical sensor could effectively measure Trp in acidic media (PBS, pH = 4) wherein the peak current for the oxidation of Trp at a chitosan-modified electrode was more than 40 time higher than that of bare GCE; see Figure 9A. This significant enhancement in sensitivity was ascribed to the proton relay effect of chitosan (Figure 9B), through hydrogen bonding between chitosan and Trp. Roushani et al. [204] went further and incorporated carbon nanodots into the chitosan film, attempting to fabricate a sensor with higher electrical conductivity and larger specific surface area. MWCNT [208], graphene oxide [209] and graphene quantum dots [210] were also mixed with chitosan and used to develop Trp sensors.

**Figure 10:**
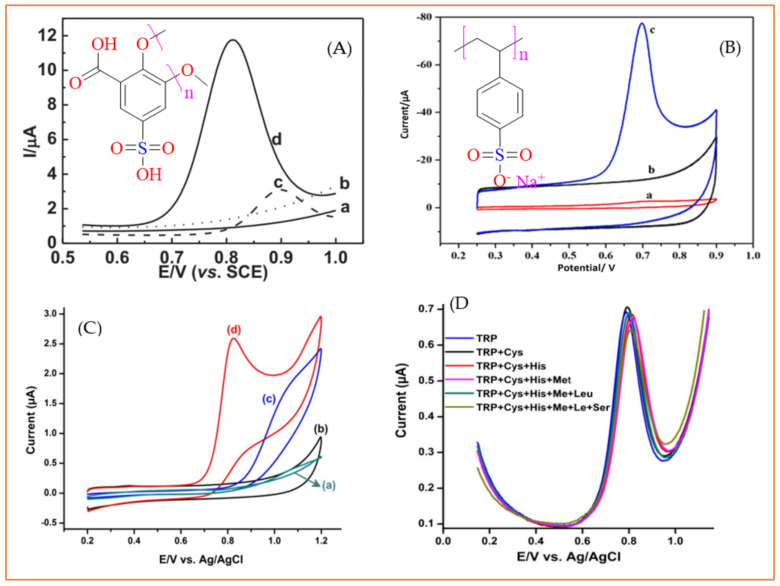
(**A**) Differential pulse voltammograms of the bare GCE (a,c) poly(sulphosalicylic acid)/GCE (b and d) in a PBS solution (0.1 M, pH = 3.5) in the absence (a,b) and presence (c,d) of 100 µM Trp. Reprinted with permission from ref. [214]. Copyright 2013 Elsevier. (**B**) Cyclic voltammograms of (a) bare GCE in PBS (pH = 6) containing 20 µM Trp, PSS–graphene/GCE in (b) blank solution (c) PBS containing 20 µM Trp. Reprinted with permission from ref. [208]. Copyright 2019 Elsevier. (**C**) Cyclic voltammograms obtained in PBS (pH 7) at bare GCE (a,c) and modified CeVO_4_/GCE (b,d) in the presence (c,d) and the absence (a, b) of 100 µM tryptophan. (**D**) Differential pulse voltammograms of tryptophan in the presence of some representative amino acids including serine (Ser), leucine (Leu), cysteine (Cys), methionine (Met) and histidine (His) Reprinted with permission from ref. [215]. Copyright 2017 Elsevier. Inset (**A**) and (**B**) are the chemical structures of poly(sulphosalicylic acid and poly(sodium 4-styrenesulphonate)), respectively.

In addition to promoting sensitivity, modifiers have also been used to suppress the interference effect of some potential interferents. Benzenesulphonic acid derivatives, for example, have been shown to successfully alleviate the interference effect of uric acid, UA and ascorbic acid (AA), especially when they are used in near-neutral or slightly acidic medium. When used, poly(sulphosalicylic acid) [211] and poly(sodium 4-styrenesulphonate) [205] caused, respectively, about 3- and 100-fold enhancement in the peak currents of Trp oxidation with respect to the bare electrode (Figure 10A,B), while, concurrently, the interference issue of UA and AA was acceptably resolved. The enhanced sensitivity is related to, on one hand, the hydrophobic interaction between the hydrophobic moiety of Trp (indole ring) and the benzene part of these modifiers and, on the other hand, electrostatic interaction between the negatively charged benzenesulphonic part and the positively charged Trp in relatively acidic medium. Additionally, the anti-interference effect against AA and UA stems from the electrostatic repulsion of negatively charged AA and UA and benzenesulphonic moiety in a slightly acidic condition. Apart from UA and AA, the presence of other amino acids can be challenging for electrochemical Trp sensors. Kumar et al. [212] successfully used a flower-like cerium vanadate (CeVO_4_) microstructure as a modifier to resolve this issue. Using this modifier in neutral medium, 0.05 M PBS pH = 7, led to an enhancement in peak current (sensitivity) (Figure 10C), whereas other electroactive amino acids, e.g., cysteine, methionine and histidine, did not show an interference effect (Figure 10D). However, the most severe interfering amino acid for electrochemical Trp detection is tyrosine, which has not been investigated by authors. 

**Figure 11:**
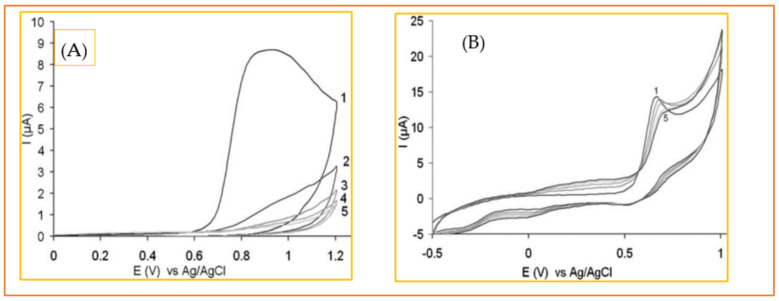
Cyclic voltammograms of 1 mM solution of tryptophan in 0.1 M PBS (pH 7) for five consecutive scans at (**A**) bare GCE and a (**B**) gold nanoparticle/ionic liquid electrode. Reprinted with permission from ref. [218]. Copyright 2010 Wiley.

Alongside the aforementioned issues, i.e., sensitivity and interference effect, electrode fouling is another challenge in electrochemical Trp sensing. This problem mainly arises from the by-products and/or products that are produced and that cover the electrode surface during electrochemical measurements. Electrode fouling or electrode passivation leads to decay in signal current over time and, consequently, the loss of the original sensitivity. Occupying the active reaction sites at the electrode surface by adsorbate species and the potential drop across the adsorbed fouling layer at the electrode surface is assumed to cause this decay in sensitivity [213]. Polymeric products, as shown in the proposed electrochemical oxidation pathway of Trp (see Figure 4) are the main suspect for electrode fouling in electrochemical Trp sensors. Figure 11A clearly shows this undesired phenomenon when a bare GCE is placed in PBS solution containing 1mM Trp, and five consecutive cyclic voltammograms are recorded. As is seen, in the first potential scan, a strong oxidation peak is observed for Trp, while the second oxidation peak declines significantly and for the next potential cycles almost no oxidation peak is detectable. Ionic liquids, i.e., molten salts whose melting point is lower than 100 °C [214], have shown great potential to face this challenge. Figure 11B shows that, in the presence of ionic liquid, just a slight decay in the oxidation peak of Trp occurs even after applying five successive potential cycles. Safavi and Momeni [215] ascribed this resistance of the electrode against fouling to the ionic liquid content of the electrode and its polarity. The ability of ionic liquid to dissolve the reaction products and drag them inside, as is seen for mercury electrodes, is assumed to play the key role. This anti-fouling capability was also reported when ionic liquids are mixed with MWCNTs [216].

Besides the aforesaid reports whose aim was mainly promoting the sensitivity of Trp sensors and, concurrently, addressing other critical issues, i.e., selectivity and electrode fouling, other reports are found in the literature that aimed at reducing the analysis time [201,217] and/or the cost [49,102] of the sensors. The reports on electrochemical Trp measurement, since 2010, were collected and tabulated and are presented in Table 4. 

#### 3.2.2. Tyrosine

Tyrosine (Tyr) is known as a non-essential amino acid since, naturally, the body can produce it from another amino acid called phenylalanine. Additionally, Tyr is found in meats, wheat, nuts, eggs and dairy products, especially in cheese, where it was first discovered. Tyr is the main ingredient in protein supplements that are commonly used to treat an inherited disorder called phenylketonuria. Note that the phrase “non-essential amino acid” should not lead to a false conclusion that the function of Tyr is not important for the body. On the contrary, Tyr is one of the most important amino acids in terms of body function and application in different industries; see Table 1. Since the level of Try, like other amino acids, is affected by different factors, e.g., gender, age, dietary habits, physical activity, etc., different values have been reported so far for the normal level of Try in biological fluids. The normal level of Try in human blood has been reported to be 48.6 ± 3.0 µM [336], 77 ± 12 µM [337], 90.6 ± 22.9 [338] and 30–120 µM [339], while for a healthy individual, free tyrosine excretion in urine could be in the range of 88–270 µmol per day [340]. However, adverse clinical manifestations of elevated Try level do not typically appear until the Tyr concentration of blood exceeds 500 µM [341].

**Figure 12:**
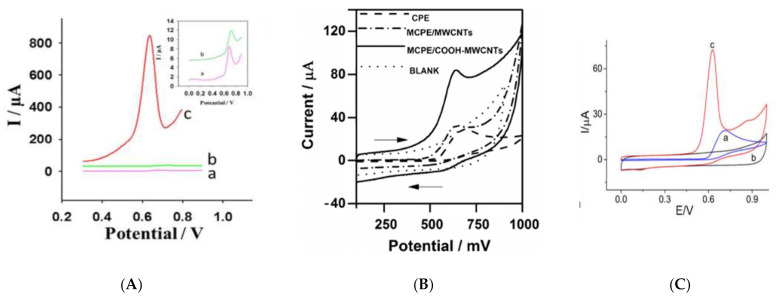
(**A**) SW voltammograms of 50 µM Tyr in 0.1 M PBS (pH 6.7) on (a) bare GPE, (b) electrochemically pretreated GPE and (c) rGO-modified GPE. Reprinted with permission from ref. [342]. Copyright 2015 Royal Society of Chemistry (**B**) Cyclic voltammograms of 500 µM Tyr in PBS (0.1 M, pH 7.0) at a CPE, modified CPE-MWCNTs and modified CPE/COOHMWCNTs at a scan rate of 50 mV s^−1^. Reprinted with permission from ref. [346]. Copyright 2016 Springer. (**C**) Cyclic voltammograms of a SWCNH-modified GCE in the absence (b) and presence (c) of 1 mM Tyr and (a) a bare GCE in the presence of 1 mM Tyr. The scan rate is 50 mV/s, and the supporting electrolyte is 0.1 M PBS (pH 7.0). Reprinted with permission from ref. [306]. Copyright 2014 Springer.

Undoubtedly, carbon-based materials are of among the most important modifiers used to construct electrochemical Tyr sensors in terms of both enhancing the sensitivity and/or selectivity of the sensor. Various typologies of carbon nanomaterials have been used so far for Tyr detection, including reduced graphene oxide [342], MWCNTs [343], SWCNTs [344], graphene quantum dots [345] and born-doped diamond [346]. Baig and Kawde [342] proposed a very cheap, simple and reusable electrochemical sensor for Tyr simply through the electrodeposition of graphene oxide on a graphite pencil electrode (GPE). Though the preparation method was quite straightforward, the resultant sensor showed an extraordinary enhancement in oxidation peak current (ca. 104 times compared to unmodified GPE), as shown in Figure 12A. The LOD of this sensor was calculated to be 0.07 µM, and it was successfully used to measure Tyr in urine samples. In another simple yet effective strategy, D’Souza et al. [343] blended MWCNTs with carbon paste electrode (CPE) to fabricate a sensor that was able to measure Tyr in a linear range of 0.8–100 µM with LOD of 0.014 µM in neutral pH. Authors reported that carboxylic-acid-functionalized MWCNTs offer a significant improvement in sensitivity compared to pure MWCNT (see Figure 12B) due to the electrocatalytic activity of the carboxylic functional groups. Single-walled carbon nanohorns (SWCNHs), as a member of CNT family, was exploited by Zhu and colleagues [303] to develop a sensor for both Try and Trp. Adding this modifier to a bare GCE led to a sharp promotion in the Tyr oxidation peak current, as shown in Figure 12C. This enhanced sensitivity is supposed to be a consequence of the significantly high surface area and edge plan-like defects of SWCNHs that provide numerous favorable active sites for electron transfer to occur. In spite of the above-mentioned carbon nanostructures, graphene quantum dots (GQD) have not yet been used alone in Tyr sensing, and they have always been combined with other modifiers such as β-cyclodextrin [345] and RuCl_3_ [347].

**Figure 13:**
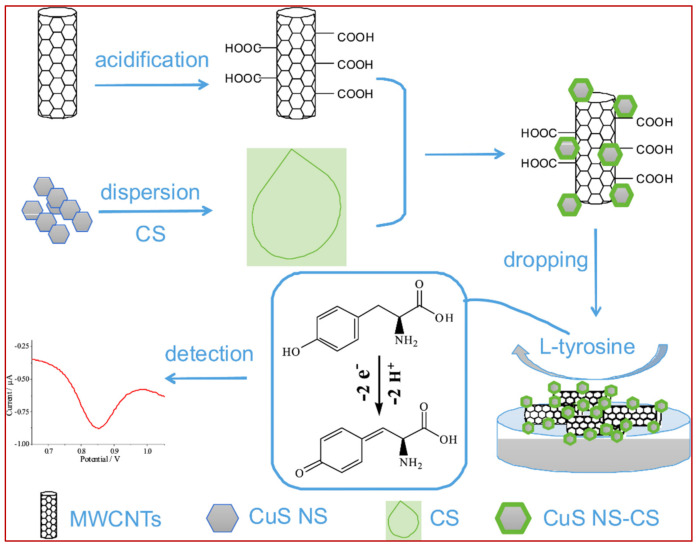
A schematic pathway of constructing the electrochemical Tyr sensor by dropping functionalized-MWCNT and CuS nanosheets in chitosan (CS) dispersions on a GCE surface. Adapted from. Reprinted with permission from ref. [351]. Copyright 2019 Elsevier.

Figure 13 represents an example of a general pathway that is extensively used to prepare electrochemical sensors. This method commonly involves the following steps. Firstly, one, two or more desired modifiers are synthesized separately by different chemical methods. Afterwards, these modifiers are mixed together in a film-forming agent such as chitosan (CS) or Nafion using ultrasonication. The resulting homogenous dispersion is then dropped on the working electrode surface and left to dry in air or in a nitrogen stream or under infrared radiation. Zhu and colleagues [348] used this relatively simple method by mixing functionalized MWCNT and copper sulphide (CuS) nanosheets in chitosan (CS) and dropping 4 µL of this dispersion on a glassy carbon electrode (GCE) surface. This electrochemical sensor (CuS/MWCNT/GCE) showed a quite effective function with an obtained linear measurement range of 0.08–1.0 µM and limit of detection of 4.9 nM. A selectivity test revealed that even a 50-fold higher concentration of methionine, histidine and other 12 non-electroactive amino acids showed no interference effect. However, this sensor could tolerate an interference effect of just 2-fold of Trp concentration, and cysteine was not tested. Gu et al. [349] synthesized functionalized MWCNT by acid treatment, as was done by [348], and used copper oxide nanoparticles instead of CuS to fabricate a Tyr electrochemical sensor (CuO_x_/MWCNT/GCE). This sensor was used to measure Tyr in the linear range of 0.2–200 µM. Additionally, this sensor was claimed to be insensitive to tryptophan even when its concentration is 50-fold higher than Try. D’Sousa et al. [350] has also reported an amperometric sensor based on a MWCNT/poly-2, 6—dichlorophenolindophenol film modified electrode that is able to measure Tyr in the presence of a 10-fold excess of Trp.

Of electroactive amino acids, methionine and histidine are usually reported to show negligible interfering effect on Tyr measurement, wherein cysteine [351,352,353] and, especially, Trp [316,354] are the main concerns. To resolve the peak overlapping issue, both new numerical methods and sensing materials have been proposed so far. Ghoreishi and Malekian [280] proposed a numerical solution named multivariate curve resolution-alternating least squares (MCR-ALS) to resolve the overlapped peaks of Tyr and Trp and quantify these two amino acids simultaneously. The proposed strategy was successfully applied to measure Tyr and Trp in the linear range of 0.4–175.0 µM and 0.1–200.0 µM with LOD as low as 0.1 µM and 0.04 µM, respectively. Karimi and Heydari [265] synthesized mesoporous silica nanoparticles and incorporated them into a carbon paste electrode (MSNs/CPE) to be used for the simultaneous determination of Tyr and Trp. However, this proposed electrochemical sensor suffered from severe overlapping of Tyr and Trp oxidation peaks. To alleviate this deficiency, a clustering-of-variables concept based on partial least squares (PLS) regression models was proposed, and the developed sensor was used to measure Trp and Tyr in an artificial urine sample. Tashkhourian and coworkers [291] approached this issue by developing a so-called H-point standard addition method to resolve overlapping oxidation peaks of Tyr and Trp.

**Figure 14:**
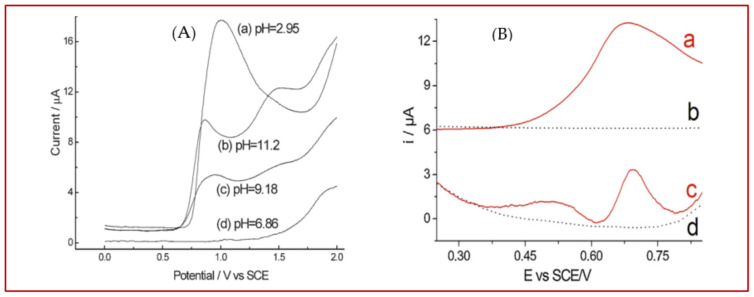
(**A**) Differential pulse voltammograms (DPVs) of Tyr and Trp using a BDD electrode in PBS with different pHs. Reprinted with permission from ref. [356]. Copyright 2006 Wiley. (**B**) DPVs of a mixture containing 2.4 mM Cys and 0.4 mM Tyr in PBS (0.1 M, pH = 7.4) at GCE (a) and OMC–GCE (c) where dotted lines present DPVs of GCE (b) and OMC–GCE (d) in a blank solution (PBS 0.1 M, pH = 7.4). Reprinted with permission from ref. [95]. Copyright 2013 Elsevier.

In addition to mathematical solutions, a few successful attempts have also been reported to address this issue by altering the sensing materials and conditions. In a very simple and quite effective strategy, Zhao et al. [355] showed that overlapped oxidation peaks of Tyr and Trp can be completely separated into two distinct peaks at an unmodified boron-doped diamond (BDD) electrode simply by changing the pH of solutions; see Figure 14A. They reported that, in acidic medium (pH = 2.95), oxidation peaks of Tyr and Trp emerge as an overlapped peak, while no oxidation peak was observed in a neutral pH of 6.86. However, in alkaline media with pH = 9.18 and especially pH = 11.2, oxidation peaks of both Tyr and Trp were clearly observed at 1.50 V and 0.86 V (vs. SCE), respectively. In contrast to [355], Deng et al. [309] designed an electrochemical sensor by mixing an acetylene black paste electrode with graphene for the simultaneous determination of Tyr and Trp, and the best performance was observed in an extremely acidic medium (1 M sulphuric acid). When the concentration of H_2_SO_4_ was less than 0.4 M, the oxidation peaks of Tyr and Trp were fully merged and resulted in a broad overlapped peak. Yocus et al. [282] modified a GCE using a similar methodology as shown in Figure 13, i.e., they synthesized two modifiers separately, dispersed them in a solution and then dropped 15 µL of that solution onto a GCE. Taking advantage of rGO and polyoxometalate, as modifiers, not only separated the oxidation peaks of Tyr and Trp but also resulted in an ultrahigh-sensitivity sensor, which was able to detect Tyr and Trp in the linear range of 0.01–1.0 nM with a LOD as low as 2.0 pM (2.0 × 10^−12^ M). This extraordinarily high sensitivity was attributed to the higher electroactive surface area of the modified electrode and the synergistic effect of rGO and polyoxometalate, wherein rGO shows high electrical conductivity, and polyoxometalate offers excellent redox properties and electron relaying effect. Zhou et al. [95] tried to resolve the overlapping oxidation peaks of Tyr and Cys using ordered mesoporous carbon (OMC) as a sensing material. As shown in Figure 14B, at bare GCE, a single oxidation peak (at 0.67 V) is observed for both Cys and Tyr; it is split into two well-resolved peaks at 0.49 V and 0.69 V on the OMC-modified electrode. This differentiating effect is related to the very high electroactive surface area of OMC that carries numerous oxygen-containing functional groups. These functional groups are able to change the reaction pathway and favor an electrochemical reaction over other possible pathways. Trypan blue, an azo dye, was electropolymerized on the GCE surface and then decorated through the electrodeposition of gold nanoparticle to be used as an electrochemical senor to measure Tyr and Cys simultaneously. Taei and colleagues [53] reported that proposed sensor can resolve the overlapped oxidation peaks of these amino acids and detect them in the range of 5.0–270 µM (for Cys) and 0.5–880.0 (for Tyr). The presence of abundant phenolic and amine functional groups on Trypan blue was likely responsible for this oxidation peak separation. In spite of very few reports, as mentioned above, it seems that this issue, i.e., the simultaneous determination of electroactive amino acids, is still challenging and needs more attention since, in many real samples, both biological fluids and industrial products, these amino acids occur together.

Table 5 summarizes the most important figures of merit for reported Tyr electrochemical sensors (since 2010) to give a panoramic view to readers concerning the latest achievements and orientations in this field.

### 3.3. Basic Amino Acid

#### Histidine

As another essential amino acid for human and other mammals, histidine (His) must also be supplied from dietary sources. Owing to the presence of an imidazole functional group (see Figure 3), histidine shows some matchless properties compared to other amino acids that are of vital importance for body function. This imidazole moiety of His, pK_a_ 6.0, is partially protonated at physiological pH and is commonly involved in many enzyme-catalyzed reactions through the proton shuttling effect [391]. His exists throughout all human tissues and has been detected in most biofluids, such as urine, blood and sweat. For normal individuals, the concentration of His in blood plasma is reported to be in the range of 70–125 µM, while the His level in urine is in the range of 52–162 (µmol/mmol creatinine) according to [392] or 130–2100 µmol/L, as reported in [393]. Comparatively, the electrochemical analysis of histidine in urine is preferred over blood samples, since urine sampling is easier, safer, non-invasive, and generally, urine samples contain less potential interference. Moreover, monitoring the urinary level of histidine is of clinical importance for some serious disorders, such as histidinemia [394].

**Figure 15:**
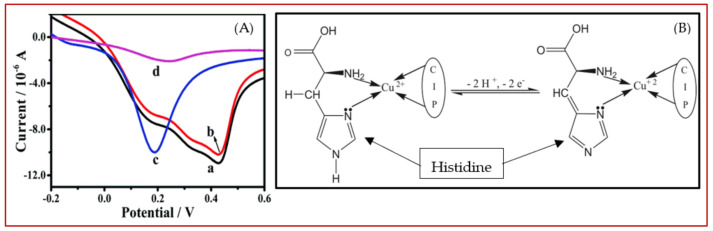
(**A**) Comparison of the linear sweep voltammograms for Cu-modified electrodes in the (a) absence and (c) presence of Cl^−^ ions, after the addition of His (b) and (d). Reprinted with permission from ref. [400]. Copyright 2019 Royal Society of Chemistry. (**B**) The proposed structure of the complex formed between Cu (II) and histidine. Reprinted with permission from ref. [403]. Copyright 2011 Elsevier.

Among all electroactive amino acids we mentioned so far, histidine shows the lowest electroactivity insofar as some researchers consider it as a non-electroactive amino acid [395]. Because of this weak electroactivity in aqueous solution, very few papers are found on the electrochemical detection of histidine (see Table 6). This lower electroactivity can be seen either as a challenge or as a great opportunity to devise new strategies for the electrochemical detection of histidine, as there is plenty of room for improvement. Hua et al. proposed a novel sensor for histidine based on the solid-state electrochemistry of copper chloride [396]. Their strategy involves following the oxidation signal of CuCl in a solution containing chloride ions after the gradual addition of histidine. They found out that the presence of Cl− is critical for the proper function of this sensor. As Figure 15A shows, in the absence of chloride ions in the test solution, oxidation peak current of CuCl remained almost unchanged after addition of Histidine to the solution (Figure 15A, curves a and b). However, if Cl− is added to the test solution, the oxidation current of CuCl decreased significantly, i.e., turn-off sensor upon the addition of histidine (Figure 15A, curves c and d). This sharp decrease in the solid-state CuCl signal is mainly due to the stronger Cu–His interaction, thanks to the imidazole group of His, compared to CuCl. This electrochemical sensor could be used in urine samples and detects His at concentrations as low as 0.025 pM. Noteworthy, the same group proposed another sensor for His that worked based on the same principals, CuCl solid-state electrochemistry, wherein the oxidation peak current of CuCl increased upon the addition of His (turn-on sensor) [397,398]. This sensor was also very selective for His, and, apart from lysine and cysteine, other tested species did not show an interference effect.

Focusing on Cu electrochemistry, Parsad et al. [399] reported that Cu (II) ions in a complex imprinted polymer (CIP) could trigger the electroactivity of His where no electroactivity was observed in the absence of Cu (II) ions. Authors supposed that this enhancement in His electroactivity in the presence of Cu (II) is related to the bond formation between Cu (II) and the amino and imidazole groups of His, as shown in Figure 15B. This complex formation between Cu (II) (present inside the CIP) can enhance the electroactivity of His through the inhibition of tautomerization (–NH–C N– ↔ –N C–NH–) in the imidazole ring of His.

Stepping away from copper as the key element, some researchers have tried other alternatives to construct electrochemical His sensors. Zhang and coworkers [400] aimed at improving both the sensitivity and selectivity of His sensors by using functionalized MWCNT and a molecular imprinted film on an ITO electrode. The developed sensor exhibited a limit of detection as low as 2.0 µM and ability to detect His in human blood serum. Staden [401] has proposed a potentiometric sensor that could detect His in a very wide linear range of 10^−5^ to 10^−11^ mol/L. In this electrochemical sensor, a fullerene-based compound played the key role as the sensing part. Interestingly, fullerene-based modifiers used in this study also showed enantioselectivity features. d-His isomer showed a stronger electrochemical signal over l-His, since the complex formed by d-histidine with the fullerene-C70 compound is more stable than that of l-histidine. This sensor was successfully used to measure d-His in pharmaceutical samples. The same author reported another potentiometric His sensor using maltodextrins as the sensing element [402]. The applicability of this potentiometric sensor was compared to capillary electrophoresis, wherein both methods showed almost the same performance for His determination. Among metal oxides, NiO [403,404] and Co_3_O_4_ [405] have been evaluated as His sensors so far; the function of these electrochemical sensors is mainly related to the electrocatalytic activity of these metal oxides in alkaline media (e.g., 0.1 M NaOH).

Not surprising, due to the very weak electroactivity of His, almost all developed electrochemical His sensors so far rely on an intermediate element, e.g., CuCl, NiO, Co_3_O_4_, fullerene etc., to resolve the very low electroactivity issue of His. Therefore, very few reports are found that mentioned the direct oxidation of His at a bare electrode [406]. The developed electrochemical sensors for His, from 2010 to date, were collected and compared with respect to their important figures of merit in Table 6.

**Table 6:** Proposed electrochemical histidine sensors, since 2010, along with the most important corresponding figures of merit.

Sensing Part	Method	LDR	LOD	L.T. Stability	Real Sample	Ref.
graphene quantum dot-scaffolded melamine and copper nanocomposites	LSV	0.1 pM ^24^–70 µM	0.025 pM	-	urine	[396]
tetrahedral copper metal organic framework	LSV	0.1–200 µM	0.025 µM	˃6 months	human blood	[397]
reduced copper metal-organic framework	SWV	0.010–100 µM	0.002 μM	˃12 months	red wine and urine	[398]
polydopamine Decorated Co_3_O_4_/rGO	AMP	10–260	1.5	-	l-His supplement	[405]
dl-homocysteine functionalized fullerene-C_60_-gold nanocomposite	SWV	0.01 pM–100 µM	1 fM ^25^	82% after 25 days	bovine serum albumin	[407]
copper germanate nanowires	CV	5–2000	1.3	-	-	[408]
hourglass-like nickel hydroxide nanostructure	CV	0.1–500	0.08	-	blood serum	[403]
nickel hydroxide nanostructures	CV	0.1–100	0.013	-	blood serum	[404]
complex imprinted polymers	ASDPV ^26^	9.99–323.6 ng/mL	1.98 ng/mL	90% after one month	bharmaceutical and blood serum	[399]
MIP/MWCNTs	DPV	2–1000	5.8 nM	-	human blood serum	[400]

^24^ Pico mol/L (= 10^−12^ mol/L); ^25^ Femto mol/L (= 10^−15^ mol/L); ^26^ Anodic stripping differential pulse voltammetry.

## 4. Conclusions: Challenges and Opportunities

Thanks to many attractive analytical features, electrochemical sensors represent promising candidates for future clinical and even point-of-care diagnostic tests. Many of the electroanalytical approaches presented in this review allow an inexpensive, straightforward, rapid and highly sensitive analysis of five electroactive amino acids and even proteins containing at least one of these amino acids [409,410,411,412], without involving preconcentration and derivatisation step(s). However, apart from these prominent features, there are some major challenges to overcome before electroanalytical approaches become approved, more than what they are today, for real-world applications. The most critical remaining issues as follows:

The first issue in the electroanalysis of amino acids is the inactivity of many amino acids on common bare carbon electrodes like glassy carbon electrodes, screen-printed carbon electrodes and born-doped diamond and carbon paste electrodes. Indeed, tryptophan, tyrosine and cysteine usually show well-defined oxidation peaks, whereas methionine and histidine electrooxidation results in, if any, a relatively weak oxidation peak. Apart from the five abovementioned amino acids, other amino acids show almost no detectable voltammetric signal in aqueous solutions. Exploiting electrochemical methods like potentiometry [402] or electrochemical impedance spectroscopy (EIS) might be the solution, since these methods do not require electroactive analytes to work. On the other hand, some literature mentioned that the addition of some substances can trigger the electroactivity of amino acids, e.g., Cu (II) for the detection of histidine [399].

The second challenge in the electroanalysis of amino acids arises from the lack of selectivity in the electrochemical detection of amino acids. It is mostly reported that electrochemical methods cannot differentiate a d-isomer from a related l-isomer of a given amino acid. More challenging, even the oxidation peak potentials of electroactive amino acids themselves are very close, and, hence, recognizing amino acids in a mixture is hard to achieve. To solve this difficulty, researchers have proposed some effective strategies. Molecularly imprinted polymers (MIP) [346] and the cyclodextrins family [413] were successfully used to alleviate this issue by adding extra selectivity to electrochemical methods. This strategy is based on the shape-recognition ability of these materials. They can be successfully used to separate one amino acid from another and even to distinguish an l-isomer of an amino acid from its d-enantiomer. Besides, chemometrics methods, e.g., partial least squares (PLS) regression [265] and multivariate curve resolution-alternating least squares (MCR-ALS) [280], are being used in electrochemical analysis to alleviate the selectivity challenges.

The third critical issue in the electrochemical detection of amino acid that electroanalysts encounter is electrode fouling due to the adsorption of reagents, products and/or intermediate species during electrochemical analysis. This drawback prevents the prolonged use of an electrode and brings about difficulties in using electrochemical detection in the continuous monitoring of amino acids. This issue is mostly encountered when electrochemical amperometric detection is coupled with chromatography, capillary electrophoresis and flow-injection analyses [414]. To overcome this issue, different approaches have thus been developed. One of the most interesting solutions is applying innovative pulsed potential sequences with three [415], four [416], five [417] and even six steps to clean and/or regenerate the electrode surface. Additionally, ionic liquids are reported to greatly prevent electrode fouling and passivation when they are used to construct an electrode [215,216]. Additionally, flow-injection analysis (FIA) is another approach to alleviate electrode fouling since the electrochemical measurements are carried out in a continuous flowing carrier stream. During the measurement, the analyte of interest is alternately injected to the carrier stream. Here, once electrochemical measurement for an injection is done, the electrode is exposed to the pure carrier stream (without the analyte) in order to wash away the by/products produced during the electrochemical measurements and to provide a clean surface for the next injection. Moreover, coating the electrode with special polymers such as nafion, chitosan, poly(ethylene glycol), poly(vinyl chloride) and polypyrrole might be used to prevent the fouling agent from reaching the electrode surface and thereby reduce the electrode passivation [418].

Looking backward, although acceptable progress has been made in the electrochemical sensing of amino acids, further advances are still needed, mainly based on developing new materials and methods. Looking forward, thanks to unprecedented efforts devoted by researchers all over the world, there is great optimism that the previously mentioned challenges can be overcome in the upcoming years.

## Figures and Tables

**Table 1:** Highlighted functions of electroactive amino acids. (Note that the first column represents structures at neutral pH).

Amino Acid	Body Function Importance	Industrial Importance
Cysteine 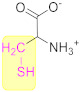	✓ Being amphoteric, generally, amino acids can act as a biological buffer. ✓ Sulphydryl side chain of cysteine is known as a strong metal binder and, then, is frequently used in metal proteins to fix their metals in place. ✓ Being able to form disulphide bridge, cysteine residues stabilise the three-dimensional structure of proteins. ✓ Cysteine is used in the body to produce a strong antioxidant named glutathione. ✓ When needed, the body uses cysteine as a source of energy through converting cysteine to glucose. ✓ Cysteine is important in communication between immune cells [7] ✓ Cysteine is abundantly found in structural proteins, e.g., keratin and collagen ✓ WHO announced the amount of 4 mg/kg of body weight per day as estimated cysteine requirement for a healthy adult [8]	✓ In the food industry, cysteine is used as antioxidant to preserve fruit juice and as an additive to flour to enhance the kneading of the dough and as a processing aid for baking. ✓ Cysteine may be used in preparations to alleviate skin lipid production and acne, as well as in anti-dandruff shampoos. ✓ It is used in selective protein purification due to its appreciable reactivity at neutral pH and low abundance in intracellular proteins [9]. ✓ In pharmaceutical industry, cysteine is extensively used as antidote to counteract toxicity of other components, e.g., acetaminophen. ✓ In the cosmetics industry, cysteine is replacing thioglycolic acid owing to its ability to break the disulphide bond in keratin in haircare products. ✓ Cystine, formed from two cysteines, is widely used to produce nail-care products since it improves fingernail growth and hardness [10].
Methionine 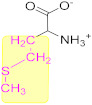	✓ Used as a methyl group donor to synthesis different organic compounds, e.g., alkaloids ✓ Preventing fatty liver through choline formation and transmethylation ✓ Alleviation of the toxic acetaldehyde level after alcohol digestion in human ✓ Preserving the cell membrane structure [7] ✓ In organisms, methionine is used as a precursor to produce amino acid cysteine. ✓ Methionine takes part in glutathione metabolism. ✓ As was mentioned for cysteine, methionine plays an important role in nourishing hair, nail and skin, as well as acetaminophen detoxification. ✓ The recommended allowance of sulphur-containing amino acids for a healthy adult is estimated to be 13 mg/kg per day [11], and its estimated requirement, recommended by WHO, is 10 mg/kg per day [8].	✓ In the food industry, methionine is an additive to improve the nutritional quality of human food or animal feeds, e.g., methionine added to soybean as pig feed. ✓ Nowadays, a large amount of chemically produced methionine in the world is used in animal feed for livestock production. ✓ In the food industry, methionine is as antioxidant used in the preservation of milk powder, as well as a nutritive element for infant milk and in sports supplements production. ✓ Methionine is used in the pharmaceutical industry in hepatic therapeutics drugs and to prevent hepatic impairments [12].
Tryptophan 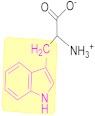	✓ Tryptophan is used as a substrate to produce plant hormones, e.g., indole acetic acid and vitamins B3 and B6. ✓ Tryptophan affects circadian rhythms through its role in the serotonin and melatonin production pathway. ✓ Tryptophan also plays role in the synthesis pathway of niacin, NAD/NADP and tryptamine [13]. ✓ The recommended allowance for a healthy adult is estimated to be in the range of 3.5–6 mg/kg of body weight per day [14].	✓ Feed industry, e.g., as a feed additive, especially for weight gain in livestock production ✓ Food industries, e.g., as an essential nutrient in fortified infant foods, corn tortillas and dietary supplements ✓ Pharmaceutical industries, e.g., in sedative and antidepressant medicines for schizophrenia treatment [15]. “Tryptan” is a tryptophan-based drug prescribed for patients as an alternative to conventional antidepressants.
Tyrosine 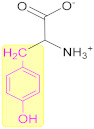	✓ In the body, tyrosine serves as a substrate to produce hormones, e.g., epinephrine, norepinephrine and thyroid hormones (T3 and T4), skin pigment melanin and neurotransmitters like dopamine. ✓ Tyrosine contributes to the synthesis of body’s natural pain-relieving agents, e.g., enkephalins. ✓ The WHO-recommended amount of (tyrosine + phenylalanine) for a healthy adult is 14 mg/kg per day. (Note that tyrosine pairs with phenylalanine to form an amino acid pair.) [8]	✓ In food industries, tyrosine is used as a flavouring agent. ✓ It is widely used in common dietary supplements, intended to act as appetite suppressant, to improve memory and to control depression and anxiety [16]. ✓ Tyrosine is used in the synthesis of *p*-hydroxycinnamic acid and *p*-hydroxystyrene, two key components in the production of advanced polymers, biocosmetics, adhesives and coatings, and nutrition products [16] ✓ Pharmaceutical industries, e.g., as a precursor for the production of high-value compounds like Levodopa as an anti-Parkinson’s disease medication [17] ✓ It is an important precursor in the synthesis of flavonoids and alkaloids as a widely used compound in food, pharmaceutical and cosmetics industries [18].
Histidine 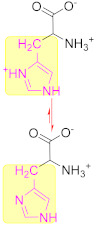	✓ It is used to produce histamine in the body. ✓ It is found in the active site of enzymes and facilitates the formation and breaking of bonds. ✓ Histidine is the only amino acid that, due to pka 6.0 of its side-chain, can switch between protonated and unprotonated forms in neutral pH and thereby can shuttle protons in many cellular enzymatic reactions [19]. ✓ Histidine participates in a broad spectrum of physiological processes such as inflammation, neurotransmission, allergic responses, the synthesis of hemoglobin and gastric acid secretion [20]. ✓ In nervous system, histidine take an important role in maintenance of myelin sheaths that protect neurons. ✓ WHO announced 8–12 mg/kg per day as an estimated histidine requirement for a healthy adult [8].	✓ In the food industry, histamine is used, e.g., as an antioxidant for the preservation of milk powder. ✓ Histidine is the first limiting amino acid for cow milk protein production, and hence it is necessary to be added to the feed when cows are fed cereal-based and grass silage supplements [21] ✓ In the harmaceutical industry, it is widely used as a component in nutritious products for infants and adults [22].

**Table 2:** Proposed electrochemical cysteine sensors, since 2010, along with the most important respective figures of merit.

Sensing Part	Method	LDR ^1^ (µM)	LOD ^2^ (µM)	L.T. Stability ^3^	Real Sample	Ref.
screen-printed diamond electrode	CV	1–194	0.62	-	bovine plasma	[124]
Co–La oxides/rGO composite	AMP	1–888	0.1	-	serum and commercial syrup	[125]
Mn-La oxides/reduced graphene oxide composite	AMP	0.5–832.5	0.1	93.2% after one week	commercial serum and syrup samples	[126]
cobalt hydroxide nanosheets	AMP	0.2–1940	0.0765	-	human blood serum	[127]
silver metal-organic frameworks coated onto nitrogen-doped porous carbon	LSV	0.1–1300	0.05	˃6 months	milk sample	[128]
CuO/Boron Nitride Nanocomposite	AMP	1–10	0.58	98% after 25 days	blood serum	[129]
Co_3_O_4_ nanoparticles	AMP	0.2–75	0.07	-	urine sample	[130]
functionalized MWCNT	DPV	0.7nM-200µM	0.16 nM	˃one week	blood serum	[131]
graphite-polyurethane composite	AMP	30–130	4.24	-	food supplements	[132]
3D pothole-rich hierarchical carbon framework-encapsulated Ni nanoparticles	CV	0.8–85	0.15	90% after one week	Blood serum and urine	[133]
copper pentacyanonitrosylferrate and octa(aminopropyl)silsesquioxane	AMP	200–2000	125	-	-	[134]
Cu^2 +^-phen-dione@rGO	AMP	10–32,344	2	˃15 days	urine sample	[135]
ferrocene-functionalized mesoporous silica	CV	3–20	3	-	-	[136]
CeO_2_-CuO nanocomposite	AMP	10–5000	0.16	93.7% after 5 days	pond water	[137]
Au–Cu@Cu_x_O	AMP	1.25–1940	1.25	˃5 weeks	human blood serum	[138]
Pd@Ti_3_C_2_T_x_ nanocomposite	AMP	0.5–10	0.14	˃one week	urine sample	[139]
CeO_2_-SnO_2_ nanocomposite	AMP	10–2000	0.016	˃4 days	pond water	[140]
CuFe_2_O_4_/rGO–Au composite	CV	50–200	0.383	93% after 4 week	urine sample	[141]
Co(II)–Al(III) layered double hydroxide	DPV	10^−4^–1	10^−4^	-	pharmaceutical samples	[142]
fluorinated cobalt phthalocyanine and ordered mesoporous carbon	DPV	20–20,000	1	90% after 2 weeks	cell lysate, human serum and urine	[143]
ethyl 2-(4 ferrocenyl [1-3]triazol-1-yl) acetate/graphene	SWV	4.0–2300.0	0.9	-	blood serum and urine	[144]
free-standing TiO_2_ nanotube	AMP	100–10,000	100	93% after 80 days	human serum sample	[145]
bacteriophage particles-carbon nanofiber	CV	20–1000	20	-	-	[87]
cobalt-poly (naphthylamine)/sodium dodecyl sulphate	AMP	1–100	0.8	˃5 weeks	human urine	[146]
Au nanoparticles/ anthraquinone-2-carboxylic acid	DPV	15–500	1.873	92.5% after 2 weeks	blood serum	[49]
electrodeposited copper/SPE	AMP	1–1800	0.21	-	human and rabbit blood serum	[147]
imine substituted cobalt(II) phthalocyanine	CV	0.01–0.10	0.003	-	human urine and cysteine tablet samples	[148]
molecularly imprinted Prussian blue-porous carbon-CNTs/polypyrrole	DPV	1.0×10–z^7^–0.1	6.01 pM	94.42% after 15 days	blood serum	[112]
poly(*p*-coumaric acid)/MWNT	DPV	7.5–1000	1.1	-	human urine	[88]
Cu^2+^ modified Fe_3_O_4_@polydopamine	SWV	0.010–500	83.0 pM	-	blood serum	[113]
polyaniline/zinc bismuthate	AMP	50–2000	0.19	-	-	[149]
^4^ RGO/Nafion film decorated Pd nanoparticles	AMP	0.5–10	0.15	92.27% after 5 days	human urine	[67]
silver-copper sulphide	AMP	1–100	0.24	Should be kept in dark condition	dietary supplement	[106]
Fe_3_O_4_@NiO magnetic nanoparticles	DPV	0.1–120	0.014	89% after one month	human breast milk, cow milk and honey	[150]
Co-Gd_2_O_3_ nanocomposite	AMP	1–100	0.23	92.3% after one month	milk, cysteine capsule	[151]
bismuth tellurate nanospheres	CV	0.1–2000	0.46	˃2 weeks	-	[152]
hollow cubic Cu_2_O particles/Nafion	CV	5–200	0.4	96% after 10 days	amino acid injections	[105]
MgO nanoparticle/acetylferrocene	DPV	0.1–7000	0.03	95% after 25 days	urine, pharmaceutical serum	[79]
Au/CeO_2_ nanofibers composite	AMP	2–200	0.01	90% after one week	blood serum	[102]
CeO_2_ nanofibers	AMP	2–200	0.02	-	blood serum	[76]
Prussian blue	AMP	100–600	67.4	-	-	[114]
MnO_2_-TiO_2_ nanocomposite/2-(3,4 dihydroxyphenethyl) isoindoline-1,3-dione	SWV	0.025–200	0.013	94% after one month	blood serum, urine, cysteine capsule	[153]
CuO–Cu_2_O heterojunction	^5^ PEC	0.2–10	0.05	˃6 weeks	urine sample	[77]
ZnO nanoparticle/N-doped RGO	AMP	0.1–705.0	0.1	-	cysteine capsule	[86]
nickel oxide NPs/N-doped RGO	AMP	0.3–1620.8	0.1	93.1% after 7 days	syrup sample	[81]
thiolated catechol	AMP	0.12–34.6	0.0605	-	urine sample	[89]
Li-doped bismuth oxide nanorods	CV	0.1–2000	0.17	-	blood serum	[154]
Au-nanoparticles/poly-Trypan Blue	DPV	5–270	0.006	-	blood serum and urine	[50]
carbon black functionalized with syringic acid	CV	20–1000	0.639	-	chicken flesh and blood serum	[90]
Impurity-containing carbon black	AMP	50–700	0.0459	-	blood serum	[51]
bismuth nickelate nanorods	CV	0.5–2000	0.087	-	-	[155]
gold nanoparticles incorporated polypyrimidine derivative	DPV	2–500	0.02	94.9% after one month	blood serum and urine	[52]
CdSe quantum dot-modified/MWCNT ^6^ hollow fiber	DPV	0.287–33,670	0.116	-	bodybuilding supplements	[156]
magnetic CoFe_2_O_4_/SiO_2_ spinel-type	DPV	0.02–425	0.2	70% after one week	milk sample	[115]
MIP ^7^/CuNPs/nonporous gold	DPV	0.5–10,000 nM	70 nM	95.4% after one week	bovine serum and sauce of instant noodle samples	[71]
Y_2_O_3_ nanoparticles/nitrogen-doped RGO	AMP	1.3–720	0.8	-	syrup sample	[85]
polythiophene layer sensitized anatase TiO_2_	PEC	100–800	12.6	93.9% after 2 weeks	-	[83]
Pt-Fe_3_O_4_/RGO	AMP	100–1000	10	94.2% after 2 weeks	-	[116]
polypyrrole/graphene quantum dots@Prussian Blue	AMP	0.2–1000	0.15	-	cysteine tablets	[117]
polydopamine-capped silver nanoparticles	LSV ^8^	0.05–300	0.023	˃2 months	blood serum	[68]
Au nanoparticles/poly(E)-4-(*p*-tolyldiazenyl)benzene-1,2,3-triol	DPV	2–540	0.04	-	human urine	[53]
thulium hexacyanoferrate	DPV	0.5–8.92	0.016	91% after one week	human urine, river water	[157]
zinc bismuthate nanorods	CV	0.1–2000	0.074	˃2 weeks	-	[158]
Ru(III) Schiff base complex, multi-walled carbon nanotubes and Nafion	AMP	50–500 mg/L	0.11 mg/L	-	pharmaceutical products	[159]
Co(II)-phthalocyanine	SWV	2.6–200	4	-	embryo cell culture	[160]
Fe(II)-exchanged zeolite	SWV	0.005–300	0.00015	˃8 months	human serum, urine and *N*-acetyl cysteine tablets	[161]
molybdenum nitride nanosheets/N-doped MWCNT	CV	5–12,600	3.64	92.9% after 2 weeks	blood serum	[162]
carbon ionic liquid electrode with terpyridine copper(II) complex	CV	0.1–40	0.01	93.4% after 15 days	blood serum	[107]
zirconium (IV) phosphate/Ag hexacyanoferrate (III)	AMP	10–80	10.2	-	-	[163]
bare glassy carbon electrode	CV	1–10	0.03	-	-	[164]
V-substituted polyoxometalates/Au@2Ag core–shell nanoparticles	AMP	0.025–7.625	0.0276	90% after 2 weeks	milk sample	[72]
Fe_2_O_3_ nanoparticles supported on N-doped graphene	AMP	0.2–400	0.1	90.6% after one week	syrup sample	[78]
Au NPs-Ni-Al layered double hydroxide composite	DPV	10–1000	6.0	79.3% after 4 days	-	[73]
GO/CCNTs/AuNPs@MnO_2_^9^	DPV	0.01–7.0	0.0034	-	human urine	[55]
bismuth nanostructure incorporated into ionic liquid	SWV	1–2000	0.5	-	blood serum	[165]
silver nanoparticles modified hierarchically structured ZnO	PEC	0.67–34.77 pM ^10^	0.21 pM	94.1% after one month	human urine	[69]
cyclotricatechylene	CV	0–20	0.9	-	cell tissue media	[166]
(DMBQ) ^11^/ZnO nanoparticles	SWV	0.09–340.0	0.05	˃one month	urine, water and pharmaceutical serum	[167]
iron tetrasulphonated phthalocyanine decorated MWCNT	AMP	10–200	1	95.16% after one month	blood serum	[118]
copper inorganic-organic hybrid coordination compound	AMP	10–80	2.1	-	-	[108]
14-(4-hydroxyphenyl)-14-*H*-dibenzo[a,j]-xanthene/MWCNT	DPV	4–1000	1	-	human serum, acetyl cysteine tablets	[123]
gold nanoparticle (AuNP)-iron(iii) phthalocyanine	DPV	50–1000	0.27	-	pharmaceutical sachets, dietary supplement	[56]
Sulphonated Graphene-poly(3,4-Ethylenedioxythiophene)/Au NPs	AMP	0.1–382	0.02	95.25% after 10 days	human urine	[57]
bismuth film	SWV	1–10	0.028	-	dietary supplement	[168]
carbon ionic liquid	LSV	1–450	0.298	-	artificial urine and nutrient broth	[91]
polyaniline/CuGeO_3_ nanowire	CV	1–2000	0.44	-	-	[109]
nanocarbon (carbon black)	SWV	0–100	5	-	-	[94]
SnO_2_–MWCNTs	AMP	0.1–554.5	0.03	92% after one month	-	[82]
MWCNTs/gold NPs stabilized with calcium cross-linked pectin	AMP	0.1–1000	0.019	-	blood serum	[58]
methacrylic acid based MIP	DPV	0.02–0.18	0.0096	-	blood plasma, tapwater samples	[169]
protoporphyrin/WO_3_/RGO	PEC	0.1–100	0.025	93.74% after 4 weeks		[84]
MWCNTs/ gold nanorods	AMP	5.0–200	0.0085	˃3 months	blood serum	[59]
graphene nanosheets/manganese oxide nanoparticles	AMP	1–24	0.075	-	-	[170]
MnO_2_ nanoparticles	AMP	10–640	0.8	-	blood serum	[171]
dispersion of MWCNTs in metallopolymer	AMP	0.025–0.151	0.006	˃one month	-	[119]
Ce-doped Mg–Al layered double hydroxide	AMP	10–5400	4.2	92.7% after 2 weeks	syrup sample	[172]
gold nanoparticles	CV	1–14 pM	0.6 pM	-	-	[61]
Co(II)-exchanged zeolite Y	CV	1 nM–1 mM	0.24 nM	˃9 months	blood serum, urine, *N*-acetylcysteine tablet and powdered poultry feed samples	[173]
nanoporous gold	AMP	1–400	0.05	-	human urine	[62]
Au-NPs/poly-eriochrome black T	AMP	0.05–100	0.008	95% after 10 days	-	[63]
graphene oxide/Au nanocluster	CV	0.05–20	0.02	87.3% after 12 weeks	human urine	[64]
titanium (IV) phosphate composite	CV	200–9000	334	-	-	[174]
benzoylferrocene-modified MWCNTs	SWV	0.7–350	0.1	-	human hair, *N*-acetylcysteine tablet	[97]
vertically aligned MWCNTs modified with Pt nanoparticles	AMP	1–500	0.5	-	human urine	[103]
caterpillar-like manganese dioxide–carbon nanocomposite	AMP	0.5–680	0.022	-	˃one month	[175]
Au NPs dispersed in Nafion	AMP	4.0–80.0	1.0	-	-	[65]
cobalt hexacyanoferrate NPs with a core-shell structure	AMP	3–37	0.04	-	blood serum and urine samples	[39]
yttrium hexacyanoferrate nanoparticle /MWCNT/Nafion	AMP	0.2–11.4	0.16	-	-	[176]
CdS quantum dot-methyl viologen complex	PEC	0.2–2.8	0.1	-	-	[177]
silver pentacyanonitrosylferrate	CV	0.1–20	0.035	-	˃three months	[121]
gold-aminomercaptothiadiazole core-shell NPs	AMP	0.01–0.14	3 pM	96.5% after 10 days	blood serum and urine samples	[178]
conducting polymer/Au NPs	AMP	0.5–200	0.05	-	l-cysteine capsule	[66]
*p*-aminophenol/MWCNTs	DPV	0.5–100	0.3	-	urine, river water, blood plasma and serum samples	[98]
electrospun carbon nanofibers	AMP	0.15–64	0.1	-	-	[99]
1,1′-Ferrocenedicarboxylic acid	DPV	20–500	9.8	-	water samples	[122]
AuPt alloy/MWCNTs-ionic liquid	AMP	0.5–40	-	95% after 15 days	-	[74]
quinizarine	DPV	1–1000	0.22	-	blood serum, acetylcysteine tablet	[100]
copper hexacyanoferrate	AMP	1–13	0.13	-	human urine	[110]
Pt nanoparticles/poly(o-aminophenol) film	AMP	0.4–630	0.08	93% after one month	syrup sample	[104]
gallium nitride nanowires	CV	0.5–75	0.5	86% after 5 days	-	[179]
silver nanoparticles coated polyquercetin	CPM ^12^	0.1–90 nM	0.03 nM	-	-	[70]
CuGeO_3_ nanowire	CV	1–1000	0.9	˃2 weeks	tapwater	[111]

^1^ Linear dynamic range in µmol/L (unless otherwise specified); ^2^ Limit of detection in µmol/L (unless otherwise specified); ^3^ Long-term stability; ^4^ Reduced graphene oxide; ^5^ Photoelectrochemical; ^6^ Multi-walled carbon nanotube; ^7^ Molecularly imprinted polymer; ^8^ Linear sweep voltammetry; ^9^ Graphene oxide/carboxylated multiwalled carbon nanotube/manganese dioxide/gold nanoparticles composite; ^10^ Pico mol/L (= 10^−12^ mol/L); ^11^ 8,9-dihydroxy-7-methyl-12*H*-benzothiazolo [2,3-b]quinazolin-12-one; ^12^ Chronopotentiometry.

**Table 3:** Proposed electrochemical methionine sensors, since 2010, along with the most important respective figures of merit.

Sensing Part	Method	LDR ^13^	LOD ^14^	L.T. Stability ^15^	Real Sample	Ref.
3D-printed electrodes	SWV	5–230	1.39	-	human serum	[196]
silver oxide	AMP	60–500	0.42	-	blood serum	[181]
single layer MoS_2_	PEC	0.1–1000 nM	0.03 nM	˃28 days	blood serum	[185]
Mn_2_O_3_	DPV	1–610	0.001		seafood sample	[187]
ruthenium/platinum bimetallic monolayer coated on a nanoporous gold film	DPV	0.006–102	0.002	97.3% after 3 weeks	human urine	[182]
Ag–Au core-shell bimetal nanoparticles	AMP	50–1000	30	96% after a week	-	[183]
ZnS/ZnAl_2_S_4_ nanocomposite	SWV	0.05–800	0.01	-	blood serum and urine	[197]
RGO/α-cyclodextrin	AMP	170–1200	40	˃7 days	-	[192]
graphene oxide	DPV	450–4950	100	-	-	[193]
imprinted polybenzidine/MWCNTs functionalized –COOH	DPV	11.7–206.3 ng/L	3 ng/L	-	pharmaceutical and blood serum samples	[41]
Pt doped TiO_2_ NPs/CNT	AMP	0.5–100	0.1	88% after 2 weeks	blood serum	[189]
benzoylferrocene modified MWCNTs	SWV	0.1–200	0.058	-	urine sample	[198]
electropolymerized functionalized triazole polymer	AMP	0.1–100	4.1 × 10^−4^	97.65% after 2 weeks	urine sample	[199]
electropolymerized film of non-peripheral amine substituted Cu(II) phthalocyanine	DPV	50–500	0.027	˃one month	blood serum	[190]
bare screen-printed graphite electrodes	DPV	50–5000	95	-	pharmaceutical products	[194]
cobalt hydroxide nanoparticles	AMP	245–1210	160	-	-	[191]
MWCNTs	AMP	360–6900	270	-	pharmaceutical product	[195]
fullerene-C_60_ modified gold electrode	CV	Up to 100	8.2	-	root beer syrup and methionine pill	[200]

^13^ Linear dynamic range in µmol/L (unless otherwise specified); ^14^ Limit of detection in µmol/L (unless otherwise specified); ^15^ Long-term stability.

**Table 4:** Proposed electrochemical tryptophan sensors, since 2010, along with the most important corresponding figures of merit.

Sensing Part	Method	LDR (µM)	LOD (µM)	L.T. Stability	Real Sample	Ref.
octahydropyrimido[1, 2-a] azepine	DPV	1.5–750	0.05	˃one month	blood and urine samples	[218]
nickel nanoparticle/Nitrogen-carbon nanohybrid	SDLSV ^16^	0.01–80	0.006	92.77% after 2 weeks	human serum and pharmaceutical samples	[219]
3-neomenthylindene	DPV	2.5–300	1.71	95.4% after 5 days	urine and blood plasma	[220]
CeO_2_/rGO composite	SDLSV	0.01–10	0.006	88.7% after 2 weeks	amino acid injection, human serum and urine	[221]
CeO_2_/rGO composite	DPV	0.2–25	0.08	˃one month	milk and bovine serum samples	[222]
silver zeolite nanocomposite	DPV	0.01–1.2	0.0063	-	wheat flour, goat and cow milk	[223]
pencil graphite electrode	ASDPV ^17^	0.154–200	0.046	-	urine sample	[224]
rGO decorated with 18-crown-6 and gold nanoparticles	SWV	0.1–2.5	0.05	77% after 50 days	human serum	[225]
poly (3,4-proplenedioxy thiophene)@nitrogen-doped carbon hollow spheres composites	DPV	0.1–100	0.0092	-	-	[226]
rGO/gold nanoparticles	DPV	0.5–500	0.39	-	saliva, serum and plasma	[227]
polythiophene/silver dendrites composite	SWV	0.2–400	0.02	-	soybeans extract	[228]
MWCNT and molecularly imprinted polymer	SDLSV	0.002–100	0.001	91% after 2 weeks	human serum	[229]
polyvinylpyrrolidone functionalized graphene	SDLSV	0.06–100	0.01	88% after 20 days	urine, serum and injection samples	[230]
CuSn(OH)_6_ microsphere decorated on rGO	DPV	0.05–175.8	0.002	95% after 15 days	urine sample	[231]
Pd-Ag nanoparticles	DPV	0.1–1000	0.1 (at pH 4)	-	-	[232]
polydopamine/rGO/MnO_2_ composite	AMP	1.23–303.26	0.24	94% after one month	tomato fruit and juice	[233]
molecularly imprinted copolymer/ MWCNT	DPV	0.008–26	0.006	92.7% after 30 days	amino acid oral liquid and human serum samples	[234]
nitrogen-doped ordered mesoporous carbon	CV	0.5–200	0.035	95.4% after one month	amino acid cocktails	[235]
rGO, gold nanoparticles, poly-l-cysteine, and poly-l-phenylalanine methyl ester	AMP	100–800	44	87.7% after 5 weeks	-	[236]
perovskite-type SrTiO_3_ nanocubes/rGO	AMP	0.03–917.3	0.0071	96.2% after 2 weeks	urine and blood serum	[237]
hydroxyapatite/graphene oxide	LSV	7–1000	5.5	-	sunflower and pumpkin seeds	[238]
poly(3,4-ethylenedioxythiophene)	CV	10–400	7.2	-	urine and serum samples	[239]
3D nitrogen-doped reduced graphene oxide and self-assembled polysaccharides	DPV	10–5000	0.0035	96.9% after 22 days	human urine and serum	[240]
silver molybdate/rGO	AMP	0.002–146.9	0.0057	-	milk and oat samples	[241]
molecularly imprinted chitosan film	SDLSV	0.01–100	0.008	91% after 20 days	human serum, amino acid injections	[242]
graphene functionalized with 3,4,9,10-perylene tetracarboxylic acid and chitosan	DPV	1–10 mM	1.2	96.7% after 2 weeks	human urine and serum	[243]
Ta_2_O_5_-rGO	SDLSV	1–800	0.84	-	human serum	[244]
MWCNT@polydopamine composite loaded with copper(II)	DPV	1–100	0.15	93.4% after 30 days	-	[245]
exfoliated graphene and poly (3,4-ethylenedioxythiophene):poly (styrene sulphonate)	DPV	0.1–1000	0.015	98.9% after 2 weeks	-	[246]
cuprous oxide and electrochemically rGO	SWV	0.02–20	0.01	94.28% after 2 weeks	human serum and commercial amino acid injections	[247]
poly(sodium 4-styrenesulphonate) functionalized graphene	LSV	0.04–10	0.02	89.2% after 7 days	human serum sample	[205]
manganese cobaltite entrapped rGO	AMP	0.004–112.9	0.001	-	milk sample	[248]
Fe_3_O_4_/C composite	SDLSV	1–800	0.26	90.4% after one week	human blood serum	[249]
gold nanoparticles electrodeposited onto graphite-polyurethane	DPV	0.6–2.0	0.053	-	synthetic urine and commercial poly-amino acids supplement	[250]
anionic/cationic-pillar [5]arenes multilayer film	DPV	1–300	0.3	-	blood serum	[105]
alumina/graphene/Cu hybrid	DPV	1–1000	0.009	˃10 days	urine sample	[251]
flowerlike Fe_3_O_4_@NiO magnetic nanoparticles	DPV	0.1–120	0.014	89% after one month	human breast milk, cow milk and honey	[150]
NiO/carbon nanotube/PEDOT ^18^ composite	DPV	1–41	0.21	100.5% after 37 days	blood serum	[252]
amino-modified β-cyclodextrin (NH_2_-β-CD), gold-platinum core-shell microspheres	DPV	10–5000	4.3	91.7% after 15 days	milk samples	[253]
surface-confined chromium-salen complex	EIS ^19^	4–60 nM	0.78 nM	-	blood serum	[254]
functionalized carbon black/poly-l-histidine nanocomposite	AMP	0.025–125.0	0.008	92% after 2 weeks	milk and human urine	[206]
Schiff-based Cu(II) complex	CV	7–48	0.185	˃3 weeks	milk sample	[255]
activated MWCNTs Ionic Liquid	CV	5–1000	2.3	-	commercial amino acid injection and blood serum	[256]
silver nanodendrites implemented in polylactide-thiacalix[4]arene copolymer	DPV	0.1–100	0.03	90% after 6 weeks	tryptophan sedative medication	[257]
cerium-doped ZnO and functionalized MWCNTs	DPV	0.01–0.1	0.001	97% after one week	blood serum and milk samples	[258]
tetrabutylammonium bromide on the ß-cyclodextrin incorporated MWCNTs	DPV	1.5–30.5	0.07	90% after 20 days	blood serum	[259]
magnetic coreshell manganese ferrite nanoparticles/ionic liquid	SWV	5–400	1.1	-	urine sample	[260]
Pd−Cu@Cu_2_O/N-rGO	DPV	0.01–40.0	1.9 nM	-	urine and milk samples	[261]
bismuth sulphide/sulphur doped graphene nanocomposite	DPV	0.01–120	0.004	-	-	[262]
poly(l-arginine)/rGO and gold nanoparticles	DPV	0.01–100	0.1	95% after 2 weeks	urine sample	[263]
MWCNTs-CTAB ^20^ nanocomposite	DPV	4.9–64.1	1.6	-	blood serum	[264]
mesoporous silica nanoparticles	DPV	0.05–600	0.011	-	artificial urine	[265]
Fe_3_O_4_ magnetic nanoparticles/graphene quantum dots	DPV	0.08–150	0.08	-	-	[266]
nanoporous carbon	AMP	1–103	0.03	93.7% after 3 weeks	amino acid injection, fetal calf serum samples	[102]
rGO decorated with polypyrrole nanofibers and zinc oxide-copper oxide	DPV	0.053–480	0.01	97.86% after 2 weeks	blood serum	[267]
Ni-doped Lewatit FO36 nano ion exchange resin	DPV	4–560	0.38	89.7% after 2 months	water, urine, serum and pharmaceutical samples	[268]
tricobalt tetroxide nanoparticles decorated carbon nanofibers	AMP	0.005–40	0.002	96% after 20 days	pharmaceutical samples	[269]
rGO decorated with SnO_2_–Co_3_O_4_ nanoparticles.	DPV	0.02–6.0	0.0032	95.7% after 14 days	blood serum, urine and pharmaceutical samples	[270]
amino-functionalized graphene quantum dots/β-cyclodextrin	DPV	1–30	0.65	94%after 2 months	-	[207]
unzipped MWCNT incorporated overoxidized poly(*p*-aminophenol)	DPV	5–1265	0.47	94.7% after one month	blood serum and urine samples	[271]
AgNPs/graphene oxide-poly(l-arginine)	DPV	1–150	0.122	-	urine sample	[272]
graphene oxide/NiO nanocomposite and *n*-hexyl-3-methylimidazolium hexafluoro phosphate	SWV	5–700	1	-	urine and pharmaceutical samples	[273]
mixed oxide SiO_2_/Nb_2_O_5_/ZnO metallization with iron(III) and inserted into the porphyrin ring	SWV	10–70	3.28	-	pharmaceutical samples	[274]
polythiophene nanostructures	LSV	6–180	0.61	-	blood serum and urine	[275]
graphite-like carbon nitride nanosheets	LSV	0.1–110	0.024	91.8% after 30 days	amino acid injection and rat blood serum	[276]
flower-like cerium vanadate	DPV	0.1–94	0.024	98.4% after one week	milk and urine samples	[212]
rGO) decorated with SnO_2_	DPV	1–100	0.04	˃2 weeks	milk and amino acid injection samples	[277]
poly(β-cyclodextrin)/carbon quantum dots composite	DPV	5–270	0.16	94.7% after 2 weeks	urine sample	[278]
MWCNT/ ionic liquid nanocomposite	DPV	0.5–70	0.32	-	dough sample	[279]
ZnFe_2_O_4_ nanoparticles	DPV	0.1–200	0.04	-	blood serum and urine samples	[280]
MWCNTs	DPV	0.6–100	0.065	-	blood serum	[203]
MWVNTs/1-(allyloxy)-4-nitrobenzene	DPV	0.06–40	0.007	-	blood serum, milk and pharmaceutical samples	[281]
polyoxometalate functionalized rGO	SWV	0.001–1 nM	0.002 nM	97.75% after 45 days	blood serum	[282]
CTAB/phosphotungstic acid/rGO	DPV	0.1–300	0.02	99.73%after one month	amino acids injection sample	[283]
β-cyclodextrin-platinum nanoparticles/graphene nanohybrids	DPV	50–5000	17	93.7% after one week	Trp enantiomers mixture	[284]
N-doped carbon dots/β-cyclodextrin	DPV	5–70	1.7	-	Trp enantiomers in riboflavin sample	[285]
poly(l-methionine) and graphene composite film	DPV	0.2–150	0.017	89.7% after one month	milk and blood serum samples	[286]
β-cyclodextrin modified magnetic graphene oxide	DPV	0.5–750	0.3	97.4% after 15 days	commercial amino acid preparations	[287]
tellurium nanorods	AMP	0.02–11.48	0.01	90% after 20 days	commercial amino acid injection	[288]
nickel and copper oxides-decorated graphene	SWV	0.3–40	0.1	95% after one month	blood serum and pharmaceutical samples	[289]
4-amino-3-hydroxy-1-naphthalenesulphonic acid/rGO based polymer	SWV	0.5–200	0.31	93.33% after one month	pharmaceutical formulations, human urine and plasma samples	[290]
SiO_2_	DPV	0.05–400	0.034	˃2 months	artificial urine sample	[291]
NiO nanoparticle coupled ionic liquid	SWV	0.08–350	0.04	-	urine and water samples	[292]
metal-organic framework/silver nanoparticles composite	DPV	1–150	0.14	-	urine sample	[293]
Pt/CNTs nanocomposite/ionic liquid	SWV	0.1–400	0.04	˃40 days	meat and pharmaceutical samples	[294]
nitrogen-incorporated tetrahedral amorphous carbon thin film	AMP	0.1–100	0.089	-	-	[295]
silver film loaded on carbon paper	LSV	0.1–330	0.04	93% after 2 weeks	milk sample	[296]
carbon-supported NiCoO_2_ nanoparticles	DPV	50–943.4	5.7	90.31% after 20 days	blood serum, urine and pharmaceutical samples	[297]
Cu NPs/overoxidized poly(3-amino-5-mercapto-1,2,4-triazole) film	DPV	4–144	0.16		blood serum and urine samples	[298]
Fe_2_O_3_/SnO_2_ composite	DPV	0.6–70	0.1	-	blood serum and pharmaceutical samples	[299]
nitrogen-doped graphene nanosheets/CuCo_2_O_4_ nanoparticles	DPV	0.01–3.0	0.0041	-	urine, serum and pharmaceutical samples	[300]
carbon nanodots/chitosan	DPV	Up to 90	0.09	98.4% after 3 days	blood serum	[204]
chitosan film	DPV	0.1–130	0.04	˃one week	pharmaceutical samples	[42]
graphene/silicon oxide	DPV	0.5–200	0.495	96.5% after 2 months	-	[301]
MWCNTs decorated with Nickel NPs	SWV	0.02–1.0	0.0066	95.1% after 3 weeks	milk and pharmaceutical samples	[302]
SWCNTs	LSV	0.5–50	0.05	-	blood serum	[303]
Ag-MoS_2_/chitosan	DPV	0.5–120	0.05	-	urine sample	[304]
MIP from co-electropolymerization of o-phenylenediamine and hydroquinone	DPV	0.01–1.0	0.005	-	Trp enantiomers mixture	[305]
MWCNT functionalized with 5-amino-2-mercapto-1,3,4-thiadiazole	AMP	25–300 nM	0.54 nM	-	blood serum	[306]
*p*-sulphonated calix[4]arene complex	CV	0.1–10	0.03	90% after one week	blood serum and amino acid injection samples	[307]
gold nanoparticles decorated graphene oxide nanocomposite	AMP	5–25	0.29	-	-	[308]
acetylene black/graphene	DLSV ^21^	1–100	0.06	92% after 2 weeks	compound amino acid injections and humanserum samples	[309]
*p*-phenylenediamine covalently linked with cysteamine capped cadmium sulphide quantum dots	SWV	100–500	14.74	-	beverage sample	[310]
ruthenium xanthate complex	DPV	0.25–50	0.083	˃3 days	pharmaceutical samples	[311]
boron-doped diamond (BDD) electrodes and wires	DPV	Up to 250	0.5	-	blood serum	[216]
MWCNTs	AMP	0.6–100	0.033	94.6% after one week	milk and blood serum samples	[216]
MWCNT modified sol-gel	DPV	0.2–15	0.139	-	milk sample	[312]
Au NPs/poly(alizarin red S) film	AMP	0.02–20	0.0067	-	-	[313]
carbon fiber ultramicroelectrodes	CV	50–200	16.7	-	pharmaceutical samples	[217]
MWCNT/Mg-Al-CO_3_ layered double hydroxide	LSV	3–1000	0.0068	93.7% after one week	milk and blood serum samples	[314]
poly-sulphosalicylic acid	DPV	0.05–10	0.0068	95.24% after 4 weeks	blood serum and amino acid injection samples	[211]
electrochemically reduced graphene oxide	DPV	0.2–40	0.1	91.6% after 2 weeks	-	[315]
gold nanoparticles/macroporous carbon composites	DPV	10–1000	0.024	91% after 3 weeks	blood serum	[316]
Co_3_O_4_ nanoparticles-decorated graphene	AMP	0.05–10	0.01	94% after 4 weeks	liquid Dulbecco’s modified Eagle medium and amino acid injection samples	[317]
SiO_2_ nanoparticles	LSV	0.1–50	0.036	95.4% after one week	blood serum and pharmaceutical samples	[318]
TiO_2_-graphene/4-aminobenzenesulphonic acid composite	DPV	1–400	0.3	96.08% after 20 days	human blood serum	[319]
β-cyclodextrin functionalized Fe_3_O_4_ magnetic nanoparticles	DPV	0.8–300	0.5	-	amino acid injection sample	[320]
Si-doped nano-TiO_2_	CV	1–400	0.5	90.3% after one month	amino acid injection sample	[321]
oxidation product of TRP	DPV	0.5–50	0.05	˃30 days	blood serum	[322]
cobalt(II) coordination polymer	DLSV	0.2–80	0.1	˃30 days	amino acid injection sample	[323]
binuclear manganese(II) complex	DLSV	0.1–80	0.08	˃30 days	amino acid injection sample	[324]
nano-mixture of graphite/diamond	LSV	0.1–80	0.03	-	human synthetic serum	[325]
copper hexacyanoferrate film on cysteamine-gold nanoparticle graphite-wax composite	CV	0.085–120	0.0185	˃25 days	milk sample	[326]
chemical vapor deposited MWCNTs	CV	0.001–100	0.22 nM	˃2 months	pharmaceutical samples	[327]
poly(9-aminoacridine) functionalized MWCNT	DPV	1–500	0.81	˃2 months	pharmaceutical samples	[328]
nano-structured Ni (II)/(2-amino-1-cyclopentene-1-dithiocarboxylic acid) film	AMP	0.085–43.0	0.023	-	blood serum	[329]
nafion and TiO_2_-graphene nanocomposite,	DPV	5–140	0.7	92% after 2 weeks	-	[330]
*p*-aminophenol/MWCNTs	DPV	10–300	5.7	-	urine, river water, blood plasma and serum samples	[98]
boron-doped diamond nanowires	DPV	0.5–50	0.5	-	-	[331]
dibenzo-18-crown-6 and Ni^2+^ ion	DPV	1.96–1010	0.0979	-	apple, guava, red grape juice, milk and pharmaceutical samples	[332]
gold nanoparticles	SWV	5–900	4	-	amino acid injection sample	[215]
carbon ionic liquid	CV	8–1000	4.8	96.87% after 4 weeks	synthetic amino acid mixtures	[333]
poly(methyl red) film	LSV	0.1–100	0.04	-	amino acid injection sample	[334]
gold nanoparticles (AuNPs) onto carbon nanotube (CNT) films	AMP	0.03–2.5	0.01	-	pharmaceutical samples	[335]

^16^ Second-order derivative linear sweep voltammetry; ^17^ Adsorptive stripping differential pulse voltammetry; ^18^ Poly(3,4-ethylenedioxythiophene; ^19^ Electrochemical impedance spectroscopy; ^20^ Cetyltrimethylammonium bromide; ^21^ Derivative linear sweep voltammetry.

**Table 5:** Proposed electrochemical tyrosine sensors, since 2010, along with the most important corresponding figures of merit.

Sensing Part	Method	LDR	LOD	L.T. Stability	Real Sample	Ref.
2D-MoS_2_	LSV	1–500	0.5	70% after 4 months	-	[356]
macroporous carbon embedded β-cyclodextrin	DPV	1–500	0.2	-	tapwater	[357]
double-chain Cu metal organic framework	DPV	10–90	5.8	-	-	[358]
black phosphorus nanosheets/β-cyclodextrin	SWV	10–100	4.81	-	-	[359]
CdO/SnO_2_ nanoparticle	AMP	0.1nM-10 µM	0.098 nM	-	human, rabbit and mouse blood serum samples	[360]
rGO-hemin-Ag	DPV	0.1–1000	0.03	92.1% after 15 days	urine sample	[361]
plain graphite	DPV	0.01–100	0.002	-	pharmaceutical capsule	[362]
Au nanoparticles @metal organic framework/polythionine loaded with molecularly imprinted polymer	DPV	0.01–4	0.79 nM	96.8% after 2 weeks	blood serum	[363]
ErVO_4_/MnWO_4_ heterostructure	DPV	0.08–400	0.0077	-	blood serum	[364]
2D-MoS_2_ nanosheets	CV	0–100	0.5	-	commercial food integrator	[365]
cupric oxide decorated β-cyclodextrin	AMP	0.01–100	0.0082	97% after 15 days	food sample, urine and serum samples	[366]
acetylene black paste electrode modified by oxygen-functionalized MWCNTs	SDLSV	0.04–600	0.02	92.5% after 2 weeks	milk, yogurt, beer and cheese samples	[367]
ultrathin g-C_3_N_4_/Ag layers	DPV	1–150	0.14	85.8% after 4 weeks	pharmaceutical samples	[354]
copper sulphide nanosheets modified with chitosan and acidified MWCNTs	DPV	0.08–1.0	4.9 nM	93.54% after 10 days	pig serum samples	[348]
NiO nanoparticles	DPV	0.15–450	0.1	-	urine and pharmaceutical samples	[368]
electrodeposited Cysteic acid	DPV	3.5–96	1.1	˃one month	blood serum	[369]
iron oxide nanoparticles	DPV	0.4–270	0.05	˃3 weeks	blood serum	[370]
molecularly imprinted polymer/rGO	DPV	0.1–400	0.046	90.6% after 20 days	blood serum and urine samples	[371]
MWCNT/TiO_2_	DPV	0.001–100	0.001	-	human serum albumin and bovine serum albumin samples	[336]
graphene quantum dot-β-cyclodextrin	DPV	0.1–1.5	0.03	˃6 days	-	[345]
mesoporous silica nanoparticles	DPV	0.3–600	0.049	-	artificial urine sample	[265]
lead-doped carbon ceramic	AMP	5–1458	0.77	-	pharmaceutical samples	[372]
filtered MWCNTs	DPV	25–750	8	-	plasma and whole blood samples	[373]
molecularly imprinted polypyrrole film	SWV	0.005–0.025	0.0025	94.4% after 10 days	plasma sample	[374]
poly-(diallyldimethylammonium chloride)/gold nanoparticles	CCR ^22^	0.3–10	0.01	-	-	[375]
graphene oxide/MnO_2_ microspheres/chitosan	DPSV ^23^	0.02–20	0.0083	98.2% after one month	milk and dried blood spots samples	[376]
graphene nanowalls deposited on a tantalum	DPV	8–100	0.8	˃94 days	blood serum and pharmaceutical samples	[377]
Au-nanoparticles/poly-Trypan blue	DPV	0.5–880	0.008	97.1% after 4 weeks	blood serum and urine samples	[50]
graphene-zinc oxide (ZnO/GR) nanocomposite film	DPV	1–800	0.5	-	urine sample	[378]
graphene oxide/ZnO nanocomposite	SWV	0.1–400	0.07	-	pharmaceutical serum and water samples	[379]
silver nanoparticle patterned functional liquid crystalline gel	DPV	0.2–500	0.01	95% after one month	blood serum	[380]
acetylene black and chitosan	DPV	2.5–430	0.92	90.4% after 28 days	urine sample	[381]
ZnFe_2_O_4_ nanoparticles	DPV	0.4–175	0.1	-	blood serum and urine samples	[280]
SiO_2_@Fe_3_O_4_/GR nanocomposite decorated graphene/carbon ionic liquid	DPV	1–800	0.5	-	urine sample	[382]
graphene quantum dots (GQDs) and β-cyclodextrins	CV	6–1500	0.0067	98.31% after 10 days	blood serum	[383]
polyoxometalate (H_3_PW_12_O_40_) functionalized rGO	SWV	0.01–1 nM	0.002 nM	99.03% after 45 days	blood serum	[282]
SiO_2_	DPV	0.5–600	0.15	˃2 months	artificial urine sample	[291]
MWCNT/poly (Bromocresol purple)	AMP	2–100	0.191	95.8% after 7 days	milk and blood serum samples	[384]
graphene quantum dot/RuCl_3_ nanocomposite	AMP	1–937	0.23	94% after 6 weeks	-	[347]
carboxylic acid functionalized MWCNT	AMP	0.8–100	0.014	93.1% after one week	milk and blood serum samples	[343]
phthalic anhydride functionalized chitosan/carbon nanotube film	AMP	1–800	0.3	85% after 2 weeks	blood erum	[353]
gold nanoparticles involved in 2-aminoethanethiol functionalized graphene oxide	DPV	1–20 nM	0.15 nM	98.13% after 60 days	milk sample	[385]
poly(thionine)	DPV	1–250	0.57	95% after one week	blood serum	[386]
copper oxide/cuprous oxide nanoparticles/MWCNT nanocomposite	AMP	0.2–200	0.0096	90% after 3 weeks	urine sample	[349]
MWCNT/poly-2,6-dichlorophenolindophenol film	AMP	0.3–110	0.075	93.8% after 2 weeks	blood serum and soya sauce	[350]
rGO	SWV	0.8–60	0.07	-	urine sample	[342]
SWCNTs	LSV	2–30	0.4	-	blood serum	[303]
Nafion and cerium dioxide nanoparticles	DPV	2–160	0.09	94% after 2 weeks	blood serum	[387]
Fe-doped hydroxyapatite nanoparticles	AMP	0.1–10	0.245	80% after 2 weeks	-	[388]
palladium decorated MWCNT	LSV	0.1–10 nM	0.146 nM	-	-	[389]
ordered mesoporous carbon	DPV	15–900	10	95.4% after 2 weeks	-	[95]
old nanoparticles/macroporous carbon (GNPs–MPC) composites	DPV	5–1000	0.074	87% after 3 weeks	blood serum	[316]
thiolated β-cyclodextrins	DPV	36–240	12	-	pharmaceutical samples	[351]
hemin immobilized onto the poly (amidoamine)/MWCNT	AMP	0.1–28.8	0.01	-	-	[352]
europium hexacyanoferrate film	AMP	10–600	8	-	-	[390]

^22^ real-time channel current response; ^23^ differential pulse stripping voltammetry.
